# Multivariate Patterns in Mental Health Burden and Psychiatric Resource Allocation in Europe: A Principal Component Analysis

**DOI:** 10.3390/healthcare13233126

**Published:** 2025-12-01

**Authors:** Andrian Țîbîrnă, Floris Petru Iliuta, Mihnea Costin Manea, Mirela Manea

**Affiliations:** 1 Department of Psychiatry and Psychology, Faculty of Stomatology, “Carol Davila” University of Medicine and Pharmacy, 020021 Bucharest, Romania; atibirna@gmail.com (A.Ț.); mirela.manea@umfcd.ro (M.M.); 2 “Prof. Dr. Alexandru Obregia” Clinical Hospital of Psychiatry, 041914 Bucharest, Romania

**Keywords:** mental health, factor analysis, European public policies, psychiatric hospitalization, Bartlett scores

## Abstract

Introduction: In recent decades, the burden of mental disorders has become a major determinant of population health in the European Union, generating profound clinical, socioeconomic, and institutional consequences. Despite political recognition of this silent crisis, substantial methodological challenges persist in the transnational monitoring of mental health and in linking disease burden with the resources allocated to address it. The present analysis develops a multivariate taxonomy of EU Member States from a psychosocial perspective, using an integrative quantitative approach. Methods: This cross-sectional, comparative study follows international standards for transparent and reproducible quantitative reporting and is based on 18 harmonized clinical, epidemiological, and institutional indicators collected for 27 EU Member States over the period 2014–2023. The indicators used in this study were grouped according to their position along the care continuum. Hospital-based indicators refer to inpatient activity and institutional capacity, including total hospital discharges, psychiatric admissions (affective disorders, schizophrenia, dementia, alcohol- and drug-related disorders), and hospital bed availability. Outpatient and community-level indicators reflect the capacity of systems to provide non-hospital psychiatric care and consist primarily of psychiatrist density and total specialist medical workforce. Finally, subjective perception indicators capture population-level self-assessed health status, complementing clinical and institutional measures by integrating a psychosocial perspective. After harmonization and standardization, Principal Component Analysis (PCA) with Varimax rotation was applied to identify latent dimensions of mental health. Model adequacy was confirmed using the Kaiser–Meyer–Olkin coefficient (0.747) and Bartlett’s test of sphericity (*p* < 0.001). Results: Three latent dimensions explaining 77.7% of the total variance were identified: (1) institutionalized psychiatric burden, (2) functional capacity of the health care system, and (3) suicidal vulnerability associated with problematic substance use. Standardized factor scores allowed for the classification of Member States, revealing distinct patterns of psychosocial risk. For example, Germany and France display profiles marked by high levels of institutionalized psychiatric activity, while the Baltic and Southeast European countries exhibit elevated suicidal vulnerability in the context of limited medical resources. These results highlight the deep heterogeneity of psychiatric configurations in Europe and reveal persistent gaps between population needs and institutional response capacity. Conclusions: The analysis provides an empirical foundation for differentiated public policies aimed at prevention, early intervention, and stigma reduction. It also supports the case for institutionalizing a European mental health monitoring system based on harmonized indicators and common assessment standards. Overall, the findings clarify the underlying structure of mental health across the European Union and underscore the need for coherent, evidence-based strategies to reduce inequalities and strengthen system performance at the continental level.

## 1. Introduction

Mental health is one of the most critical dimensions of global public health in the 21st century, with major implications for sustainable development, social equity, and institutional resilience. According to the Global Burden of Disease Study 2019, mental disorders are among the leading causes of global disease burden, contributing to over 14% of total years lived with disability (YLDs) worldwide [1]. Disorders such as major depression, anxiety disorders, schizophrenia, bipolar disorders, dementia, and substance use disorders affect nearly one billion people, causing considerable personal distress and contributing to broader societal challenges [2,3,4,5,6,7,8].

The COVID-19 pandemic has further exacerbated existing vulnerabilities and generated a “second pandemic” of mental suffering. The incidence of depression and anxiety increased significantly, alongside rising rates of suicidal behaviours. These effects were particularly pronounced among young people and other vulnerable groups [9,10,11,12,13,14]. The pandemic disrupted not only psychiatric care but also the overall stability of health budgets, especially in vulnerable Member States [15]. At the same time, structural societal pressures have intensified psychosocial imbalances. These pressures include economic precariousness, social inequalities, forced migration, climate insecurity, and accelerated digitalization. They unfold in a broader context defined by a global polycrisis [16,17,18,19,20,21].

The configuration of psychiatric care in the European Union has undergone profound transformations. These changes are shaped by demographic ageing and evolving diagnostic practices. They also reflect a broader shift toward community-based and integrated service models [22]. According to recent estimates by the World Health Organization and Eurostat [23,24], approximately one in six European citizens suffers from some form of mental disorder each year. The report OECD/European Union—Health at a Glance: Europe 2022 [25] estimated that mental disorders generated total direct and indirect costs of over EUR 600 billion annually, equivalent to 4% of the European Union’s GDP. These costs include productivity losses, absenteeism, long-term disability, institutional care, and premature deaths from suicide or comorbidities. The impact on health systems is also amplified by the lack of integration of mental health services, chronic underfunding, and persistent stigma surrounding psychiatric diagnosis.

The spectrum of mental disorders—from depressive and anxiety conditions to schizophrenia, addictions, and dementia—continues to increase in incidence and prevalence. These disorders are often exacerbated by complex structural stressors [26,27,28,29]. This alarming trend has been partially countered by several strategic initiatives launched at both European and national levels. These initiatives aim to address mental health in an integrated and cross-sectoral manner. These include the Joint Action on Mental Health and Well-being [30], EU Compass for Action on Mental Health [31], the EU4Health Programme [32], and the package “A comprehensive approach to mental health in the EU”, launched by the European Commission in 2023 [33]. At the same time, mental health has been integrated into the pillars of post-pandemic resilience. This integration is reflected in the Recovery and Resilience Mechanism [34], cohesion policies, and the European Green Deal [35]. These frameworks acknowledge the psychosocial dimension of the green transition. Furthermore, the WHO European Mental Health Framework for Action 2021–2025 [23] and the Action Plan for the European Pillar of Social Rights [36] emphasize the importance of social inclusion and employment for people with mental health problems. They also support prevention, early intervention, and destigmatization programmes.

However, their implementation has involved major disparities across Member States. These disparities concern access to psychiatric services, the availability of specialized medical staff, hospital infrastructure, and the degree of digitization of mental health services. In addition, the burden of mental illness remains difficult to quantify and compare across countries. This is largely due to the absence of integrative analytical tools and synthetic indicators that capture the multidimensional nature of the phenomenon.

Numerous independent reports and analyses [37,38,39,40,41,42,43] highlight major disparities between Member States. Some relate to access to psychiatric treatment and the availability of specialized staff. Others concern hospital infrastructure, the degree of digitization, and mental health literacy Furthermore, reporting practices and indicator definitions differ significantly across Member States. This heterogeneity limits the consistency of available datasets and reduces the comparability required for robust, evidence-based policy decisions. The absence of a unified framework for monitoring the performance of mental health systems limits comparative understanding of the phenomenon. It also perpetuates structural inefficiencies.

The literature reveals multiple gaps in comparative studies on mental health in the EU. Most research focuses on isolated indicators, such as suicide rates or health expenditure. These approaches rarely integrate multiple dimensions into a coherent framework. Such dimensions include the prevalence of hospitalizations for mental disorders, the human and material resources of health systems, subjective population perceptions, and associated mortality. Furthermore, few studies employ advanced multivariate statistical methods. Such methods enable the extraction of latent patterns and the systematic classification of states according to their mental health profile [44,45,46,47]. Finally, existing approaches often ignore the correlation between the intensity of mental burden and institutional response capacity. This aspect limits the potential of translational analysis for public policy formulation.

In this context, the present analysis seeks to address the long-standing inconsistency of mental health monitoring in the European Union. It does so by developing a coherent and multidimensional comparative framework. Unlike previous approaches, which focus narrowly on either the prevalence of psychiatric disorders or the allocation of system resources, our analysis integrates multiple domains. These domains include epidemiological indicators such as psychiatric hospitalizations and mortality linked to suicide, alcohol, and drug use. They also include systemic resources such as psychiatrist density, hospital bed availability, and health expenditures, as well as population-level perceptions of health status. This multidimensional integration enables researchers to identify latent patterns in complex datasets and to generate new empirical insights into the structural dynamics of mental health. It also provides policymakers with a practical taxonomy of national and regional vulnerabilities. This taxonomy can guide resource allocation, reduce disparities, and support targeted strategies to strengthen the resilience and equity of mental health systems across Europe. For this purpose, we used a PCA factor analysis applied to an extensive set of variables aggregated at the national level for the Member States of the European Union. The main objective of this research is to identify the latent structures that organize the interdependencies between indicators of hospitalization for mental disorders (such as depression, addictions, dementia, or schizophrenia), associated mortality rates (suicide, alcoholism, drug use), allocated resources (number of psychiatrists, available beds, medical expenditure), and general health and life expectancy indicators. To achieve this goal, we propose the following objectives:

1. Carry out a rigorous selection of clinically and epidemiologically relevant indicators that reflect the prevalence/severity of mental disorders and the institutional capacity for diagnosis and treatment in the Member States of the European Union.
2. Apply a principal component analysis (PCA) to reduce the dimensionality of the dataset and extract statistically and clinically significant components.
3. Define the latent dimensions underlying inter-state variability in mental health by interpreting the factor loadings associated with each component.
4. Calculate standardized factor scores for each Member State and classify countries according to the resulting mental health profiles.
5. Correlate these profiles with structural aspects of health systems (human and material resources) to assess the degree of alignment between population needs and institutional response.
6. Based on the results obtained, substantiate public policy proposals tailored to the specific context of each group of countries, aimed at optimizing mental health interventions and reducing disparities.

The analysis enabled the following research questions to be answered:

Q1. How can these components and scores be used to construct a European mental health taxonomy that would enable monitoring national performance and inform differentiated and effective public policies?
Q2. What are the implications of these findings in terms of institutional reform and future strategies for prevention, early intervention, and destigmatization in mental health?

The novelty of this research lies in applying an integrated approach to a set of indicators that are rarely analyzed together in a pan-European perspective, proposing a conceptual and empirical framework for understanding the synergies and tensions between psychiatric needs and systemic resources. By identifying the common components and structural factors that shape European diversity in mental health, the resulting findings offer a solid scientific basis for informing public policy, guiding strategic resource allocation, and supporting the development of contextualized intervention models. An original contribution to the literature on mental health governance is achieved through the development of a structural taxonomy that links population needs with resource availability and efficiency. An empirical framework is also provided to facilitate the formulation of differentiated public policies grounded in the psychosocial realities of each Member State.

To further distinguish the contribution of this study within the broader field of comparative health system analysis, our approach differs from prior multidimensional typologies that have primarily classified health systems based on financing models, governance structures, macroeconomic performance, or service-delivery arrangements. While these frameworks provide valuable system-level taxonomies, they typically do not integrate psychiatric morbidity, addiction-related mortality, institutional resource allocation, and population health perceptions into a unified analytical structure. The present PCA-based taxonomy therefore advances beyond general system classifications by focusing specifically on the latent structural mechanisms through which mental health needs and institutional capacities interact across EU Member States. Although several European initiatives—such as EU4Health, the WHO European Framework for Action 2021–2025, and various Eurostat or OECD dashboards—provide valuable descriptive information on mental health indicators, they do not offer an integrated analytical model capable of revealing the latent structures linking morbidity, mortality, system resources, and population perceptions. Existing mappings remain primarily indicator-based and are not designed to identify underlying patterns or to classify Member States according to multidimensional psychosocial profiles. The gap is addressed by proposing the first empirically derived taxonomy of mental health in the European Union, obtained through multivariate analysis. Through the extraction of latent components and their organization into a structured typology, the work advances beyond descriptive monitoring instruments and establishes a framework suited for systemic comparison, benchmarking, and differentiated policy design.

Against the backdrop of simultaneous health, economic, demographic, and climate crises, the demand for coherent, cross-sectoral strategies has intensified, revealing the need for public systems to integrate psychosocial dimensions more effectively into broader resilience and sustainability frameworks. In this sense, the research results are relevant not only from an academic perspective but also for policymakers, system managers, and European institutions seeking to build a coherent, equitable, and evidence-based framework for action in the field of mental health.

## 2. Materials and Methods

### 2.1. Study Design

A quantitative, cross-sectional and comparative design is employed, in full alignment with international standards that govern transparency and reproducibility in quantitative research. The overall objective was to identify latent configurations that define the psychiatric burden and institutional response capacity among the 27 Member States of the European Union. The methodological process was structured sequentially: (1) compilation and harmonization of comparable statistical indicators; (2) integration of multiple Eurostat datasets; (3) data validation and normalization; and (4) application of Principal Component Analysis (PCA) to identify latent dimensions.

Principal Component Analysis was selected because it offers a rigorous way to reduce a high-dimensional set of epidemiological, institutional, and behavioural indicators into a smaller number of latent components that summarize the structural relationships within the data. Unlike clustering, which groups countries directly, PCA first identifies the underlying dimensions that drive cross-national variation, providing a theoretically interpretable foundation for any subsequent typology. Compared with alternative techniques such as factor analysis or structural equation modelling, PCA is more appropriate for exploratory, data-driven contexts with limited sample size, as it does not require distributional assumptions or pre-specified covariance structures.

This integrated approach combines clinical, epidemiological, and systemic indicators to capture the complexity of mental health in Europe. The design allows for cross-national comparison and pattern recognition, ensuring coherence with recognized standards of quantitative public health research [48,49,50,51,52].

### 2.2. Variables and Data Source

The data sources were exclusively official and harmonized, extracted from Eurostat databases: “health care activities”, “causes of death”, “health expenditure and financing”, “demography”, and “health personnel.” The units of analysis were the 27 Member States of the European Union, and the period covered corresponds to the years 2014–2023, depending on the complete availability of statistical series. Data processing was carried out in accordance with European statistical standards, and missing variables were treated by listwise exclusion. The set of variables was compiled based on their relevance in the literature on population mental health, including hospitalizations for mental disorders (EXTPMENTAL), dementia (EXTPDEMENT), affective disorders (EXTPAFECT), schizophrenia (EXTPSCHIZ), alcohol dependence (EXTPALC) and substance dependence (EXTPDRUG), suicide mortality (DSUICID), alcohol abuse (DALC) and drug abuse (DDRUG), deaths from diseases of the nervous system (DSNERV), psychiatrist density (PSIHSPEC), total number of discharges (EXTPT), current health expenditure (CHE), life expectancy at birth (LEX), perception of own health (PGPH), and hospital bed density (HBTI). DSNERV was included because several neurological conditions (such as dementia, Alzheimer’s disease, Parkinson’s disease, epilepsy, and neurodegenerative disorders) are strongly interlinked with mental health outcomes at both clinical and population levels. For analytical clarity and in line with the care-continuum approach, all indicators were grouped into three conceptual categories. Hospital-based indicators capture in-patient activity and institutional capacity, including total hospital discharges and specific psychiatric admissions (affective disorders, schizophrenia, dementia, alcohol- and drug-related disorders), as well as hospital bed availability. Outpatient and community-level indicators reflect the capacity to provide non-hospital psychiatric care and include, in particular, psychiatrist density and the total specialist medical workforce. Finally, subjective perception indicators capture population-level self-assessed health status, complementing clinical and institutional measures with a psychosocial dimension. These conditions frequently present with psychiatric comorbidities (e.g., depression, anxiety, cognitive impairment, behavioural disturbances), contribute to long-term disability, and place sustained pressure on mental health and social care systems. Dementia and related neurocognitive disorders are now internationally recognized by the WHO, OECD, and the European Commission, as core components of the broader mental health and neurological burden. Although classified within neurological diseases, dementia presents a strong psychiatric profile: cognitive decline, behavioural disturbances, psychotic symptoms, mood disorders, and profound functional impairment. These conditions generate long-term care needs, increase the use of psychiatric and geriatric services, and significantly contribute to caregiver burden and social exclusion. For these reasons, EXTPDEMENT was retained as a clinically and epidemiologically relevant indicator reflecting the intersection between ageing, neurocognitive decline, and mental health vulnerability, dimensions that are highly uneven across EU Member States and therefore essential for a structural taxonomy. All variables were standardized before inclusion in the model, and their distributions were tested for univariate normality (Table 1).

The indicators were selected based on their epidemiological, clinical, and institutional relevance, in line with recent WHO Europe recommendations on assessing mental health system performance [53]. Integrating them into a coherent analytical framework allowed for capturing the complexity of the relationships between the prevalence of mental disorders, institutional capacity for intervention, and population perceptions of mental health [4,54,55,56,57,58]. In this regard, Table 1 presents the structure of the 18 variables used in the model, organized into five major conceptual categories—mortality (causes of death), use of psychiatric services (health care activities), available resources (health personnel and hospital beds), public expenditure (health expenditure) and general health indicators (demography and self-perceived health).

**Table 1:** Model variables.

Symbol	Indicator Name *	Analytical Category	Type of Measure	Original Scale/Unit **	Source (Repository Code)
DT	Causes of death—deaths by country of residence and occurrence	Epidemiological outcome	Mortality	Number of deaths	Eurostat, hlth_cd_aro [59]
LE	Life expectancy at birth	General health outcome	Population longevity	Years	Eurostat, demo_mlexpec [60]
PGPH	Perceived health: share of population reporting good/very good health	Perception indicator	Self-reported health status	Percentage of population	Eurostat, sdg_03_20 [61]
CHE	Total current health care expenditure	Resource indicator	Expenditure	Euro per capita	Eurostat, tps00207 [62]
HBTI	Hospital beds	Resource indicator	Infrastructure	Beds per 100,000 inhabitants	Eurostat, tps00046 [63]
DDRUG	Deaths due to drug dependence	Epidemiological outcome	Addictive mortality	% of total deaths	Eurostat, tps00149 [64]
DSUICID	Deaths due to suicide	Epidemiological outcome	Suicide mortality	% of total deaths	Eurostat, tps00122 [65]
DALC	Deaths due to alcohol abuse	Epidemiological outcome	Addictive mortality	% of total deaths	Eurostat, tps00140 [66]
DSNERV	Deaths due to diseases of the nervous system	Epidemiological outcome	Neurological mortality	% of total deaths	Eurostat, tps00134 [67]
TSPEC	Physicians by medical specialty	Resource indicator	Workforce capacity	Number	Eurostat, hlth_rs_spec [68]
PSIHSPEC	Psychiatrists by specialty	Resource indicator	Workforce capacity	Number	Eurostat, hlth_rs_spec [69]
EXTPT	Hospital discharges—total (A00–Z99, excl. V00-Y98, Z38)	Epidemiological outcome	Hospital morbidity	Number of discharges	Eurostat, hlth_co_disch1 [70]
EXTP-MENTAL	Hospital discharges—mental and behavioural disorders (F00–F99)	Epidemiological outcome	Psychiatric morbidity	Number of discharges	Eurostat, hlth_co_disch1 [70]
EXTP-DEMENT	Hospital discharges—dementia	Epidemiological outcome	Dementia morbidity	Number of discharges	Eurostat, hlth_co_disch1 [70]
EXTP-ALC	Hospital discharges—mental disorders due to alcohol use	Epidemiological outcome	Alcohol-related psychiatric morbidity	Number of discharges	Eurostat, hlth_co_disch1 [70]
EXTP-DRUG	Hospital discharges—mental disorders due to psychoactive substances	Epidemiological outcome	Drug-related psychiatric morbidity	Number of discharges	Eurostat, hlth_co_disch1 [70]
EXTP-SCHIZ	Hospital discharges—schizophrenia, schizotypal and delusional disorders	Epidemiological outcome	Severe mental disorder morbidity	Number of discharges	Eurostat, hlth_co_disch1 [70]
EXTP-AFECT	Hospital discharges—mood (affective) disorders	Epidemiological outcome	Affective disorder morbidity	Number of discharges	Eurostat, hlth_co_disch1

* All indicators were retrieved from official Eurostat databases and harmonized at the Member State level for the period 2014–2023. Indicators were grouped into three analytical categories: epidemiological outcomes, resource capacity measures, and perception-based indicators. ** Several indicators are reported by Eurostat exclusively as absolute counts (e.g., total hospital discharges, number of psychiatric specialists, number of deaths by cause). Their inclusion in absolute form reflects data availability constraints rather than analytical preference. As such, these variables may be partially sensitive to population size differences across Member States; this potential size bias is acknowledged and revisited in the Limitations section.

The dataset was processed in accordance with European statistical harmonization and comparability standards, with the aim of ensuring robustness and inter-state validity. For observations with missing data, the listwise exclusion method was applied, retaining only those countries and years for which the datasets were complete. The analysis was performed on a balanced panel dataset covering the 2014–2023 period for the 27 EU Member States. The unit of analysis was therefore country–year, which resulted in 270 valid observations for each variable after listwise exclusion. This ensured that the ratio of observations to variables (approximately 15:1 for 18 variables) was adequate and consistent with methodological recommendations for principal component analysis. The robustness of the data structure was further confirmed by the Kaiser–Meyer–Olkin (KMO) test (0.747) and Bartlett’s test of sphericity (*p* < 0.001).

All variables were used in their original form as reported by Eurostat. No additional transformations (such as logarithmic, Box–Cox or square-root adjustments) were applied, as preliminary inspection of distributions and standardized z-scores indicated that the dataset was suitable for PCA without requiring corrective normalization procedures. Prior to extraction, all variables were standardized to a mean of 0 and a standard deviation of 1, in accordance with standard PCA practice.

### 2.3. Integration of Datasets and Limitations

The integration of the selected Eurostat databases was conducted through a systematic process of harmonization and temporal alignment, ensuring full comparability across Member States and statistical domains. Datasets covering Health care activities, Causes of death, Health expenditure and financing, Demography, and Health personnel were merged using common country identifiers and annual time references, resulting in a unified macro-database encompassing the 27 EU Member States over the period 2014–2023. The procedure adhered to the methodological principles of the European Statistical System, preserving definitional consistency, coding integrity, and measurement coherence across data sources.

Despite the high degree of standardization, certain limitations inherent to the original datasets must be acknowledged. Variations in data completeness and reporting practices persist among countries, particularly for cause-specific mortality and hospital discharge diagnoses. Differences in diagnostic classification systems, the timing of data collection, and the reliance on survey-based indicators such as perceived health may generate minor inconsistencies in temporal comparability. Furthermore, the use of nationally aggregated data may conceal regional heterogeneity and micro-level variations in service accessibility and resource allocation.

An additional methodological caveat concerns the incorporation of some hospitalization-based indicators (e.g., EXTPT, EXTPMENTAL, EXTPAFECT) reported in absolute counts rather than population-adjusted rates. Although all variables were standardized prior to inclusion in the PCA to mitigate scale effects, residual population-size bias cannot be fully excluded. Ideally, rate-level or age-standardized indicators would further enhance cross-country comparability; however, these were not consistently available for all diagnostic subdomains and years in Eurostat, and restricting the dataset to rate-based variables would have substantially reduced its completeness and altered the stability and interpretability of the extracted factorial structure. Accordingly, standardized absolute measures are retained in this analysis, with the understanding that future studies may validate the latent dimensions using population-adjusted or age-standardized indicators when harmonized data permit such extensions.

These limitations were addressed through strict validation, cross-verification with Eurostat metadata, and listwise exclusion of incomplete series. While such measures ensure robustness and comparability, the findings should be interpreted as reflecting macrostructural relationships within European health systems rather than causal effects at the individual level. Nevertheless, the integrated dataset provides a coherent, reliable, and methodologically transparent foundation for the identification of latent patterns in mental health across the European Union.

### 2.4. Statistical Methods

The principal component analysis (PCA) method used for factor analysis assumes the existence of linear relationships between the variables included and a relative normality of distributions. These conditions were tested preliminarily during statistical processing. By default, this type of analysis may omit nonlinear interactions or subtle contextual influences, reducing the actual complexity of the phenomenon to a finite set of synthetic dimensions. Nevertheless, PCA remains a validated and frequently used tool for dimensionality reduction and identifying latent structures in multidimensional research in public health. It provides a solid basis for comparability and systemic classification [71,72,73,74].

The actual factor analysis was performed by extracting the principal components, followed by a varimax orthogonal rotation, which maximizes the variance explained by each component and facilitates the practical interpretation of the loadings. Varimax rotation was preferred because it provides an orthogonal solution that preserves the independence of the extracted components, an essential requirement for constructing a taxonomy with clearly separable dimensions. Compared with oblique rotations such as Oblimin or Promax, Varimax avoids component intercorrelations that could complicate the interpretation of factor scores and reduce their suitability for subsequent comparative analyses and clustering. Given the exploratory nature of the study and the need for a stable, parsimonious, and policy-interpretable structure, Varimax offered the most appropriate balance between statistical clarity and practical applicability. The number of components retained was based on the criterion of eigenvalue > 1 (Kaiser) [75], scree plot analysis [76], and the clinical and institutional interpretability of the extracted structures [72]. Based on these, we identified three main dimensions: Component 1—institutionalized psychiatric burden, Component 2—health system functionality and health perception, and Component 3—suicidal vulnerability and addictive behaviours. For each component, standardized factor scores (z-scores) were calculated for all 27 EU Member States, which allowed the countries to be classified according to the resulting psycho-health profiles.

The usefulness of focusing on this constellation of system features lies in their ability to capture the institutionalized manifestations of mental health and the structural capacity of national systems to respond, which are often underrepresented in studies that privilege either broad sociodemographic correlates or community-level support mechanisms. While measures of living conditions, social cohesion, or informal support networks are undeniably important, they remain heterogeneous across Member States and lack the degree of standardization necessary for reliable transnational comparisons. By contrast, the analyzed indicators (hospitalizations for major psychiatric conditions, suicide- and addiction-related mortality, psychiatrist density, health expenditures, and population health perceptions) are harmonized within Eurostat and the WHO reporting frameworks, which ensures both comparability and robustness. The resulting three-component factor structure provides a parsimonious yet comprehensive framework: it distinguishes between the institutionalized psychiatric burden (reflecting the intensity of service utilization and medical resources), the functional and perceived performance of health systems (capturing both objective investment and subjective outcomes), and the acute dimension of suicidal vulnerability linked to substance use (highlighting risks insufficiently visible through service-based measures). This tripartite structure thus serves as a theoretically coherent and practically useful basis for analysis, offering researchers a replicable tool for latent pattern detection, while enabling policymakers to differentiate between systemic, institutional, and behavioural domains of risk and to design policies that are responsive to each dimension.

High communalities and KMO (Kaiser–Meyer–Olkin) values confirmed the suitability of the dataset for factor analysis, in line with classic methodological recommendations [77,78]. Furthermore, the total percentage of variance explained by the three extracted components exceeded the 60% threshold considered adequate in applied social science studies [79,80], which supports the validity of the obtained factor structure and the relevance of the identified latent dimensions. To assess the temporal stability of the factorial structure, we additionally examined the adequacy of the dataset for each year between 2014 and 2023 by computing annual KMO values, Bartlett’s test of sphericity, and year-specific PCA models. The yearly KMO coefficients remained consistently within acceptable ranges, while Bartlett’s tests were significant for all years, confirming sufficient intercorrelation across indicators. The PCA solutions obtained for individual years showed highly similar loading patterns and component configurations when compared to the pooled model, indicating that the latent dimensions are structurally stable over time and not driven by year-specific fluctuations. This factor structure provides a synthetic and robust picture of the multidimensional distribution of mental health in the European Union. Also, it provides a methodological basis for the comparative classification of countries and for the formulation of differentiated policy proposals.

This approach provides a robust foundation for exploring systemic differences and discontinuities in European mental health policies and supports the development of strategic recommendations for reducing disparities and optimizing psychiatric services in the EU. All statistical procedures were conducted in accordance with reproducibility standards for observational quantitative research, using IBM SPSS Statistics version 25.

## 3. Results

### 3.1. Sampling Adequacy and PCA Dimensionality Checks

The results provided a comprehensive overview of the latent structure of mental health indicators in the European Union. By applying the PCA method, we extracted three principal components with eigenvalues above the threshold of 1, which together explain 77.74% of the total variance in the data. The choice of this method is justified by the ability of PCA to reduce the dimensionality of a complex set of correlated data while retaining most of the original information [81,82,83,84].

Table 2 presents the values obtained for the Kaiser–Meyer–Olkin (KMO) sample adequacy test and Bartlett’s sphericity test, used to assess the relevance of applying principal factor analysis to the set of variables included in the model.

According to the data in Table 2, the KMO (Kaiser–Meyer–Olkin) sample adequacy test value of 0.747 is considered satisfactory for applying the PCA method. Bartlett’s sphericity test is significant (χ^2^ = 9259.266, df = 153, *p* < 0.001), confirming the existence of sufficient correlations between variables and justifying the application of factor analysis. In addition to the KMO (0.747) and Bartlett’s test (*p* < 0.001), we also assessed sample adequacy relative to the number of variables. The analytical matrix includes 27 observations (EU Member States) and 18 indicators, yielding a ratio of approximately 1.6 observations per variable. Although this ratio is lower than conventional sample-to-variable heuristics proposed in the factor analysis literature, the suitability of the dataset for PCA is supported by high communalities (>0.70 for most indicators), strong inter-variable correlations, and the fact that the dataset represents the full population of EU Member States rather than a sample-based estimate. Thus, the analysis is grounded in a population-level comparative framework rather than inferential sampling. The annual evaluation of the factorial structure further supports the robustness of the model (see Table A1). Year-specific KMO and Bartlett statistics confirmed the adequacy of the data for PCA across all examined years, and the resulting components displayed consistent loading profiles, with no substantial deviations in the number or interpretation of factors. This temporal consistency suggests that the three-component solution reflects stable structural relationships among mental health indicators rather than episodic or period-specific artefacts. Overall, these results confirm that the dataset is statistically adequate and conceptually coherent for PCA, providing a robust foundation for interpreting the extracted components.

### 3.2. Component Extraction and Variance Explained

We analyzed the communities identified with the principal component method to obtain relevant information on the extent to which the variability in each mental health indicator is explained by the retained factor structure. The extracted values reflect the proportion of the total variance in each variable that is explained by the three main components (Table 3). In the context of applied social science research, communalities with values above 0.50 are generally considered acceptable, while values of 0.70 or higher indicate a robust and coherent representation of the variables in the latent factor structure. Recent methodological studies confirm the relevance of these thresholds in exploratory factor analysis, highlighting that high common variance is essential for the internal validity of the model and the interpretability of the extracted components [85,86,87]. In our analysis, most variables exceed this threshold, supporting the robustness of the constructed factorial model.

The results presented in Table 3 indicate that most of the variables analyzed are very well represented in the factorial space, suggesting a solid internal consistency of the model. Thus, the variable “total number of hospital discharges for all causes” (EXTPT) has the highest communality (0.982), almost entirely reflecting its association with the latent axes of the analysis. This is followed by “discharges for mental disorders” (EXTPMENTAL—0.963), “discharges for affective disorders” (EXTPAFECT—0.935), and “psychiatrist density” (PSIHSPEC—0.937), all highlighting an essential contribution to the first component, associated with the institutionalized burden of mental disorders. Similarly, high values of the communities for “discharges for schizophrenia” (EXTPSCHIZ—0.859), “for alcohol-induced disorders” (EXTPALC—0.914), and “psychoactive substances” (EXTPDRUG—0.829) support the integrity of this dimension and its relevance in defining aggregate clinical profiles at the national level.

In terms of resources allocated to the health system, the indicators “current health expenditure per capita” (CHE—0.762), “number of hospital beds” (HBTI—0.699), and “life expectancy” (LEX—0.707) also show high commonalities. They also show a strong anchoring in the second component, which reflects the functionality and infrastructure of the health system, as well as its perceived results. The indicator for the population’s perception of its own health status (PGPH—0.607) coherently complements this dimension and confirms the relationship between subjective perceptions, life expectancy, and resource allocations.

The third component, reflecting severe psychosocial vulnerability, is well supported by variables related to direct psychiatric mortality: “deaths associated with suicide” (DSUICID—0.716), “alcohol-related mortality” (DALC—0.594), and “drug-related mortality” (DDRUG—0.685). These values confirm the robustness of this component in capturing acute and lethal manifestations of untreated or underdiagnosed mental suffering.

In addition to the pooled PCA, we conducted a year-by-year examination of the communalities for all variables over the 2014–2023 period in order to assess the temporal stability of the factorial structure. The annual extraction values presented in Table A2 indicate a remarkably consistent pattern across the entire decade, with all variables maintaining medium-to-high communalities in each separate year. Throughout the period, the core indicators associated with hospital discharges (EXTPT, EXTPMENTAL, EXTPAFFECT, EXTPSCHIZ, EXTPALC, EXTPDRUG), psychiatrist density (PSIHSPEC), and socioeconomic burden (DT, LEX, CHE) systematically preserved high extraction values, typically exceeding the recommended threshold of 0.50. This consistency demonstrates that these indicators contribute robustly to the latent dimensions across all years, rather than being influenced by isolated fluctuations or exceptional events. Even during the COVID-19 period (2020–2021), when several health-system indicators experienced structural pressure, the communality patterns remained stable, with no variable dropping below acceptable levels. The stability of the annual communalities confirms that the latent structure extracted in the aggregated PCA is strongly supported by the year-specific analyses. This reinforces the methodological reliability of the model and validates the interpretability of the three-component solution as a structural representation of European mental health dynamics rather than a product of year-to-year variation.

As shown in Table 4, the eigenvalue criterion (eigenvalues > 1), the Cattell graph analysis (scree plot), and the cumulative proportion of explained variance led to the retention of three main components, together explaining 77.74% of the total variance in the data.

The combined application of the eigenvalue greater than one rule, scree plot inspection, and explained variance thresholds is widely recommended in the methodological literature [80,88,89,90] as a robust strategy for determining the optimal number of components, minimizing the risks of over-extraction and under-extraction.

Component 1 has an eigenvalue of 8.91 and explains 49.50% of the variance, Component 2 has an eigenvalue of 3.11 (17.29% of the variance), and Component 3 has a value of 1.97 (10.95%). The factor loadings for each component are considered significant at thresholds ≥ 0.60, in line with quantitative medical research practice. The threshold of 0.60 is frequently used in psychology and public health studies to ensure clear discrimination between dimensions [91,92,93,94].

To further assess the temporal robustness of the extracted factorial structure, we computed the total variance explained for each individual year from 2014 to 2023. The annual results, reported in Table A3, indicate a highly stable configuration across the entire decade. For every year, the first three components consistently exceeded the eigenvalue > 1 criterion and jointly explained a substantial proportion of the total variance, with values oscillating within narrow and predictable intervals. Across the period, the first component systematically accounted for the largest share of variance, reflecting the institutionalized burden of mental health (hospital discharges and psychiatrist density), while the second and third components captured, respectively, the sociodemographic and behavioural–mortality dimensions. The proportion of variance explained by each component displayed minimal fluctuations from year to year, and the cumulative variance explained by the three retained components remained closely aligned with the pooled model (approximately 75–79% depending on the year). This temporal consistency confirms that the three-component solution is not dependent on a particular year or exceptional event but reflects a stable structural relationship among the indicators included in the analysis. Even during the COVID-19 period (2020–2021), when health systems experienced considerable pressure, the relative contribution of each component remained highly similar to that of the surrounding years. Such alignment across annual PCA solutions provides further evidence that the factorial structure is intrinsically stable and that the aggregated taxonomy genuinely captures long-term patterns in European mental health systems. A detailed breakdown of the annual eigenvalues and variance explained percentages is presented in Table A3. Overall, these results demonstrate that the three-component structure provides a statistically robust and temporally stable representation of variance in European mental health indicators, supporting its use as the empirical basis for the taxonomy developed in subsequent sections.

### 3.3. Rotated Structure and Interpretation of Loadings

The interpretation of the rotated component matrix (Table 5), obtained using principal component analysis with varimax rotation, highlights the three-dimensional factor structure of the analyzed data. Each component represents a coherent and clinically relevant latent dimension of mental health in the European Union. The orthogonal rotation was applied to maximize the differences between the factor loadings and obtain a clear interpretation of each latent axis.

According to the data in Table 5, three main components can be identified. Component 1 captures the institutionalized psychiatric burden and is driven primarily by inpatient activity and specialist workforce capacity, including high levels of psychiatric hospital discharges and admissions related to affective, psychotic, alcohol- and drug-related disorders, alongside a dense concentration of psychiatrists and specialized medical staff. These associations indicate that the first component primarily reflects the intensity of psychiatric hospitalizations and the availability of specialized medical resources. These associations indicate that the first component primarily reflects the intensity of psychiatric hospitalizations and the availability of specialized medical resources.

Component 2 reflects broader health system functionality and population health performance, combining high levels of health expenditure, longer life expectancy, positive self-perceived health status, and neurological disease outcomes, alongside lower reliance on hospital bed capacity. Together, these indicators suggest a dimension linked to systemic resource allocation and overall health outcomes. Detailed loadings are provided in Table 5. This component is shaped primarily by outpatient and community-level indicators, complemented by subjective perception measures, highlighting a system-functionality axis that reflects resource configuration and perceived outcomes rather than hospital-based psychiatric care. Component 3 captures a dimension of acute psychosocial vulnerability, bringing together mortality associated with alcohol use, suicide, and drug-related behaviours. This component reflects severe behavioural and social risk factors rather than institutional capacity or healthcare performance. Detailed numerical loadings for all variables are reported in Table 5. This axis is defined by indicators that fall outside the hospital-based continuum, representing acute psychosocial vulnerability that manifests independently of inpatient care and reflects behavioural risk patterns insufficiently captured through service-based measures.

Together, these three components account for 77.7% of the total variance and provide a structured representation of the interrelationships among psychiatric morbidity, health system resources, and mortality indicators across EU Member States.

To further assess the robustness and temporal consistency of the three-component structure, we computed rotated component matrices separately for each year between 2014 and 2023. The annual loadings, presented in Table A4, show a remarkably stable factorial pattern across the entire decade. For all years, the variables associated with psychiatric hospitalizations and specialist resources (EXTPT, EXTPMENTAL, EXTPAFECT, EXTPALC, EXTPSCHIZ, EXTPDRUG, PSIHSPEC, TSPEC) consistently exhibited strong and dominant loadings on Component 1, with coefficients almost identical to those observed in the pooled model. This confirms that the institutionalized psychiatric burden remains the primary latent dimension in every year of the analysis. Component 2 maintained its structural profile across the period, with health expenditure (CHE), life expectancy (LEX), and perceived health (PGPH) showing stable positive loadings, while hospital bed capacity (HBTI) continued to load negatively. These patterns indicate that the “health system functionality and perception” dimension is temporally persistent and not sensitive to sporadic annual variations. Also, Component 3 also demonstrated consistent associations with mortality from alcohol (DALC), suicide (DSUICID), and drugs (DDRUG) across all years, confirming that this axis reflects a stable latent domain of acute psychosocial vulnerability. Despite external shocks during the COVID-19 period (2020–2021), the loading patterns did not deviate substantially from those in pre- and post-pandemic years. The year-by-year diagnostics presented in Table A1, Table A2, Table A3 and Table A4 indicate that the pandemic did not introduce any substantive disruption in the covariance patterns underlying the factorial solution. The KMO values remain within acceptable bounds throughout 2020 and 2021, Bartlett’s test is consistently significant, and the communalities preserve their magnitude and interpretability without exhibiting structural breaks or sign reversals. Similarly, the proportion of variance explained by the three retained components fluctuates only marginally during the pandemic years, with variations below one percentage point, and the rotated loadings maintain a stable alignment along the dimensions of institutional burden, system capacity, and addictions–suicide mortality. Taken together, these elements demonstrate that the factorial architecture is robust to the epidemiological shock of COVID-19 and that the pandemic did not generate a reconfiguration of the underlying associations among indicators. As such, the structure extracted from the pooled PCA appears to reflect deeper and persistent macro-institutional patterns rather than short-term perturbations linked to the health crisis.

Overall, the high degree of alignment between the annual rotated matrices and the pooled component solution provides compelling evidence for the temporal robustness of the factorial structure. This stability demonstrates that the PCA-derived taxonomy reflects long-term structural relationships among mental health indicators in the European Union, rather than being influenced by short-term fluctuations. A complete year-by-year breakdown of rotated loadings is provided in Table A4.

To facilitate interpretation, the three extracted components were assigned descriptive labels that summarize their main statistical content. These labels: institutionalized psychiatric burden (named as Component 1 reflecting high rates of psychiatric hospitalisations and specialist inpatient resources), health system functionality and health perception (named as Component 2 reflecting expenditure, life expectancy, subjective health), and suicidal vulnerability associated with addictive behaviours (named as Component 3 reflecting mortality from alcohol, drugs, suicide) should be regarded as interpretative conventions rather than definitive constructs. They are heuristic categories derived from the observed factor loadings, meant to guide the discussion of clinical and institutional implications. The following section contextualizes these components in relation to epidemiological trends, systemic resources, and European mental health policies. Overall, the rotated loadings reveal a coherent and clinically interpretable three-component structure that distinguishes institutionalized psychiatric burden from broader health system performance and addiction-related mortality, providing the analytical foundation for the subsequent comparative taxonomy.

### 3.4. Results of the KMEANS Cluster Analysis

To validate whether the three latent components derived from the PCA translate into coherent country groupings, we performed a k-means cluster analysis using the standardized factor scores for Components 1–3. The optimal three-cluster solution converged in nine iterations and displayed robust separation across cluster centres. This methodological approach is consistent with recent applications of K-means in EU-wide policy and sustainability analysis, where the algorithm has been effectively used to identify structural heterogeneity among Member States [95].

The initial configuration of the cluster solution reveals three distinct structural profiles across the latent dimensions extracted through PCA. The first cluster is characterized by an exceptionally elevated score on the institutional burden component (4.35), accompanied by a moderately high value on the addictions–suicide mortality dimension (1.15) and a near-zero position on the resource capacity axis, a configuration that suggests a group of countries where mental health systems are heavily reliant on inpatient care and where clinical severity remains pronounced despite average system capacity. By contrast, the second cluster displays a markedly different pattern, combining the highest score on the addictions–suicide component (2.92) with a substantially positive value on system capacity (1.00) and a negative loading on institutional burden (−0.64); this configuration indicates health systems that possess comparatively strong structural resources but face acute challenges related to psychosocial and behavioural mortality. The third cluster exhibits a more balanced yet distinct profile, marked by a moderate positive score on institutional burden (0.91), a positive but smaller loading on system capacity (0.65), and the lowest score on addictions–suicide mortality (−1.83), suggesting a group of countries in which clinical and psychosocial pressures are comparatively limited and where institutional reliance remains present but does not reach the levels observed in the first cluster. Collectively, these initial centres demonstrate that the three latent dimensions differentiate the data space in a consistent and interpretable manner, each cluster occupying a recognizably distinct position along the axes of institutional intensity, systemic capacity, and psychosocial vulnerability (Table 6).

The iteration history indicates that the k-means procedure converged rapidly and in a stable manner, with the cluster centres exhibiting substantial adjustments during the first two iterations (particularly for Clusters 2 and 3, where the coordinate shifts reached 2.035 and 1.738, respectively) followed by a progressive attenuation of these changes across subsequent iterations. This pattern reflects a well-behaved clustering structure in which the initial separation between centres, quantified by a minimum distance of 4.590, was sufficiently large to allow the algorithm to identify distinct and stable partitions of the data space without oscillation or boundary ambiguity (Table 7).

The configuration of the final cluster centres confirms the presence of three clearly differentiated structural profiles across the latent dimensions extracted through PCA (Table 8).

According to Table 8, the first cluster is defined by a distinctly elevated score on the institutional burden component (3.50), accompanied by a moderately positive value on the addictions–suicide mortality dimension (0.58) and a small positive loading on system capacity (0.23). This combination indicates a group of countries in which reliance on inpatient psychiatric care remains markedly above the European average, while psychosocial pressures related to alcohol, drugs and suicide are also present, albeit not to the extreme levels observed elsewhere, and where institutional resources are not deficient but do not substantially mitigate the overall clinical load. The second cluster exhibits a contrasting pattern, combining a negative position on the institutional burden axis (−0.35) with the highest score on the addictions–suicide mortality component (0.88) and a slightly negative value on the system capacity dimension (−0.12). This structure suggests mental health systems characterized by comparatively low reliance on inpatient services yet confronted with pronounced social and behavioural mortality pressures, in a context where the availability of specialized resources is insufficient to counterbalance these vulnerabilities. The third cluster occupies a markedly different region of the factorial space, with a value close to zero on the institutional burden dimension (−0.11), a slightly positive loading on system capacity (0.07), and a strongly negative score on the addictions–suicide mortality component (−0.81). This configuration denotes a group of countries in which psychosocial mortality is substantially below the European mean, institutional reliance is limited but not minimal, and system capacity is balanced, collectively forming a comparatively favourable mental health profile. Taken together, the final centres demonstrate that the three latent dimensions give rise to stable and interpretable country groupings, each defined by a distinct pattern of institutional load, systemic capacity and psychosocial vulnerability, thereby reinforcing the internal coherence and external validity of the three-cluster solution.

Distances between final cluster centres confirm a clear separation between Cluster 1 and the other groups (C1–C2 = 3.874; C1–C3 = 3.870), while Cluster 2 and Cluster 3 are closer (1.719), indicating gradational differences within the same structural continuum.

**Figure 1:**
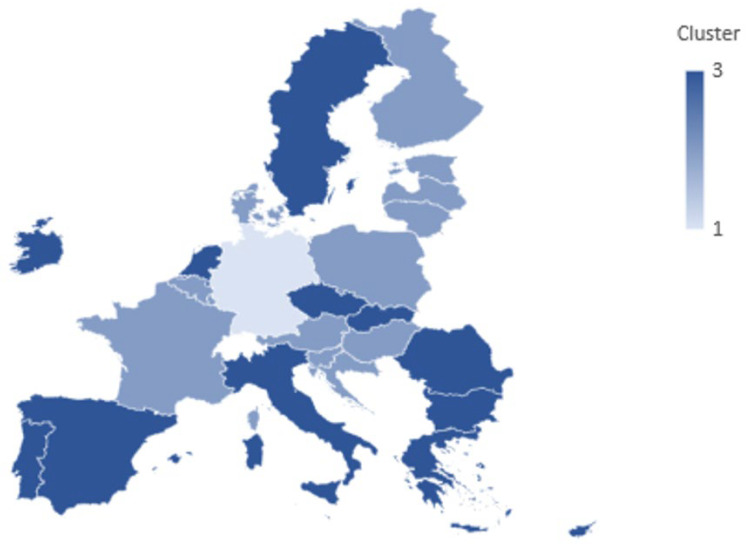
Clusters map typology of EU Member States.

Clusters typology of EU Member States based on latent mental health system dimensions are presented in Table A5. Cluster 1 contains only Germany, representing a distinct pattern of high institutionalization and high specialist concentration. Cluster 2 groups Central and Northern Member States characterized by higher expenditure, stronger system capacity, and moderate psychiatric institutionalization. Cluster 3 comprises more diverse systems, generally reflecting lower institutional reliance combined with elevated behavioural mortality risks (see Figure 1).

The ANOVA results show significant between-cluster variation for Components 1 and 3 (*p* < 0.001), confirming that the clusters reflect meaningful underlying latent structures. Overall, the three-cluster solution delineates distinct structural profiles of mental health systems across EU Member States, linking institutional reliance, resource capacity, and psychosocial vulnerability into coherent country groupings that support the interpretive taxonomy developed from the PCA.

## 4. Discussion

In discussing the three extracted components, we assigned them descriptive labels intended to capture the essence of their statistical structure. Across the three components, the indicators aligned clearly with their position along the care continuum. Hospital-based indicators clustered within the component capturing institutionalized psychiatric burden (C1), outpatient and community-level indicators together with subjective perception measures shaped the health system functionality and perception dimension (C2), while mortality indicators unrelated to inpatient care (including suicide, alcohol-related and drug-related deaths) defined the component reflecting acute psychosocial vulnerability (C3). This alignment strengthens the interpretability of the PCA structure and clarifies how each sector of the mental health system contributes to the observed latent dimensions. These labels were derived from the observed factor loadings and are meant to function as heuristic categories that facilitate interpretation, rather than as definitive constructs. Their purpose is to provide a clearer framework for understanding the interplay between morbidity, system resources, and psychosocial risks across EU Member States.

**Figure 2:**
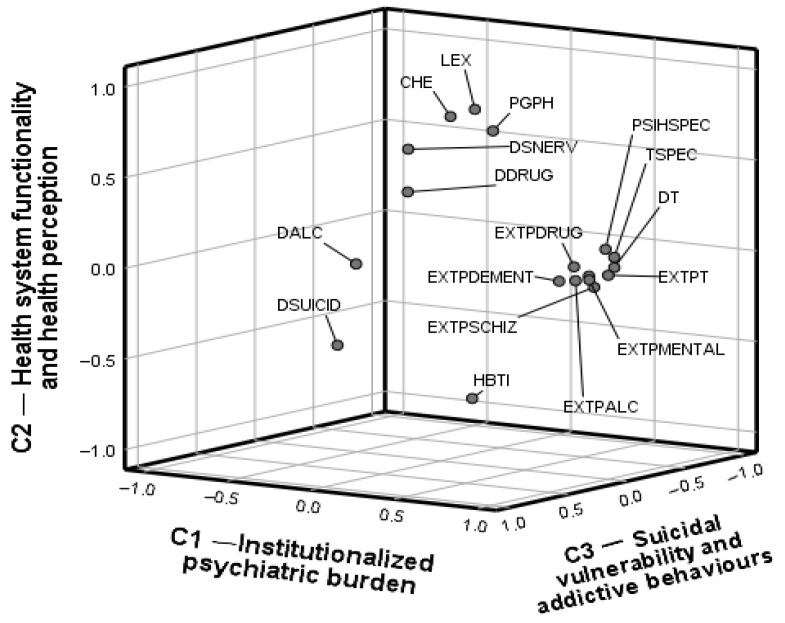
Component plot in rotated space.

The graphical representation in Figure 2 provides a coherent and relevant visual summary of the latent structure of the population psychiatry phenomenon in Europe. The three-dimensional validation of the factorial model confirms the existence of well-defined conceptual clusters with profound clinical, institutional, and public health implications. It provides a basis for further analysis of factor scores and national mental health typologies in the European Union.

Figure 2 illustrates the three-dimensional projection of the variables included in the principal factor analysis, after applying a varimax orthogonal rotation to maximize the interpretability of the factor loadings and highlight the latent dimensions that structure the interdependencies between mental health indicators at the European Union level. The three extracted components—reflecting institutionalized psychiatric burden (C1), the functional structure of the health system and health perception (C2), and suicidal vulnerability and addictive behaviours (C3)—are represented graphically as orthogonal axes that delimit a coherent factorial space with robust clinical and epidemiological significance.

The first factorial axis (C1), which defines the institutionalized component of mental health, clearly focuses on variables that quantify the frequency and diversity of hospitalizations for major mental disorders, namely, hospitalizations for affective disorders (EXTPAFECT), schizophrenia (EXTPSCHIZ), dementia (EXTPDEMENT), alcohol-induced disorders (EXTPALC), and drugs (EXTPDRUG), along with the total number of hospital discharges (EXTPT) and overall mortality (DT). The presence of the variables PSIHSPEC (density of psychiatrists) and TSPEC (total number of specialist doctors) in this component highlights the structural link between the prevalence of psychiatric diagnoses and the institutional capacity to manage them therapeutically. It also suggests a clinical axis of intensity and systematization of psychiatric care at the national level. This axis can also be interpreted as an indirect indicator of institutional maturity in mental health management, correlated with the level of specialized human resources and the capacity for organized medical response [96,97,98,99].

The first component combines indicators of both psychiatric morbidity (e.g., hospitalizations for affective disorders, schizophrenia, dementia, substance-related conditions) and systemic capacity (e.g., psychiatrist density, total discharges).

Although dementia concerns a partially distinct population group and falls under a different policy framework than affective or psychotic disorders, its inclusion in this analysis is intentional. Dementia functions as a frontier condition, positioned at the intersection of psychiatry and neurology, which justifies its integration into the present model. Dementia generates an exceptionally high consumption of institutional resources and mirrors one of the most pressing demographic pressures in Europe—the ageing of the population [100,101,102]. As such, it constitutes a critical component of the broader “institutionalized burden” of mental health, given that it requires not only hospital-based infrastructure but also extensive community and long-term care networks.

Excluding dementia-related hospitalizations from the analysis would have significantly limited the comparability between Member States with older demographic structures and those with younger populations, thereby reducing the explanatory power of the model. While we do not equate dementia with affective or psychotic disorders in terms of etiology or clinical trajectory, we regard it as an essential indicator for capturing the real pressures placed on psychiatric services and for assessing the coherence of integrated mental health policies across the European Union.

Rather than a methodological artefact, this configuration may signal a feedback mechanism between demand and supply: systems with higher burden tend to mobilize or maintain more resources, and, conversely, a stronger institutional infrastructure facilitates higher diagnostic and hospitalization rates [103,104,105,106]. Nevertheless, we acknowledge that morbidity and system capacity are conceptually distinct constructs, and their integration into a single component should be interpreted with caution. These structural patterns resonate with recent evidence indicating that psychiatric hospitalization dynamics are highly sensitive to systemic shocks and acute disruptions.

Some authors [103,104,105,106] demonstrated that psychiatric admissions exhibited marked fluctuations during both the L’Aquila earthquake and the COVID-19 lockdown, revealing the extent to which institutional capacity is stress-responsive. Their findings highlight that hospitalization levels may rise, fall, or shift diagnostically when health systems face abrupt epidemiological or social shocks, exposing underlying fragilities or resilience within institutional structures. Integrating this crisis-related perspective enriches the interpretation of our ‘institutionalized psychiatric burden’ dimension, suggesting that cross-country variability reflects not only long-term structural characteristics but also the differential ability of national systems to absorb sudden spikes or declines in psychiatric demand.

The second component (C2) expresses an integrative functional dimension of health systems, in which both objective and subjective indicators converge: current health expenditure per capita (CHE), life expectancy (LEX), and subjective perception of health status (PGPH). The positioning of these variables in the upper quadrant highlights a positive relationship between the financial inputs of the health system, overall clinical performance, and the population’s perceptions of its own health. It is noteworthy that the HBTI variable (hospital bed density) is positioned in opposition, in the lower quadrant. This suggests a model of efficiency in which volumetric infrastructure is inversely proportional to perceived performance, possibly reflecting the transition to community and outpatient care models at the expense of traditional institutionalization.

The third factorial axis (C3) configures a pathognomonic component for acute and undiagnosed forms of mental suffering, reflected in mortality associated with self-harming and addictive behaviours. The variables suicide (DSUICID), alcohol-related mortality (DALC), and drug-related mortality (DDRUG) are distinct from the other variables, signalling the exceptional and serious nature of this latent dimension. These pathologies express a high degree of mental disorganization, often outside the radar of specialized medical intervention, marking serious dysfunctions in early detection, prevention, and therapeutic integration. The third component groups suicidality and mortality related to alcohol and drug use, which can also be regarded as part of the broader disease burden. The fact that these indicators load separately from the hospitalization-based variables of Component 1 suggests that the “burden of disease” in mental health is not homogeneous. It manifests along two distinct axes: one institutionalized, observable through medical records and service utilization, and another acute and lethal, often underdiagnosed and less dependent on service capacity [107,108]. This split highlights the multidimensional nature of the psychiatric burden in Europe, where service-based measures and mortality-based measures capture different, yet complementary, aspects of population mental health [24,109,110,111,112].

The three-dimensional positioning of the variables in the factorial space also highlights indicators with mixed or transitional valence. DSNERV (mortality from diseases of the nervous system) and DDRUG (mortality induced by substance use) occupy intermediate positions between components C2 and C3. This placement suggests clinical overlaps between neurological etiology and the behavioural expression of mental disorders.

By applying PCA to indicators relevant to mental health in EU Member States, we extracted three main components. The first reflects the institutionalized psychiatric burden, characterized by high rates of psychiatric hospitalisations and substantial specialist inpatient resources. The second captures health system functionality and perceived health, including expenditure, life expectancy, and subjective health status. The third represents suicidal vulnerability linked to addictive behaviours, encompassing mortality from alcohol, drugs, and suicide.

It should also be noted that the interpretation of country-specific factor scores may be influenced by population size, as several hospitalization indicators are reported in absolute counts. Although standardization mitigates this effect, a residual size-related bias cannot be fully excluded, particularly for large Member States. These components reflect institutional, structural, and behavioural aspects, providing a nuanced overview of how different national systems respond to the challenges of mental disorders [17,113,114,115]. The additional year-by-year validation of the PCA confirms that the latent structure identified in the pooled model remains stable across the 2014–2023 period.

This reinforces the interpretability of the taxonomy as a structural representation of European mental health patterns, rather than an outcome of short-term fluctuations or exceptional events.

### 4.1. Classification of European Union Member States According to Psychosocial Risk Patterns Identified with Principal Component Analysis

**Figure 3:**
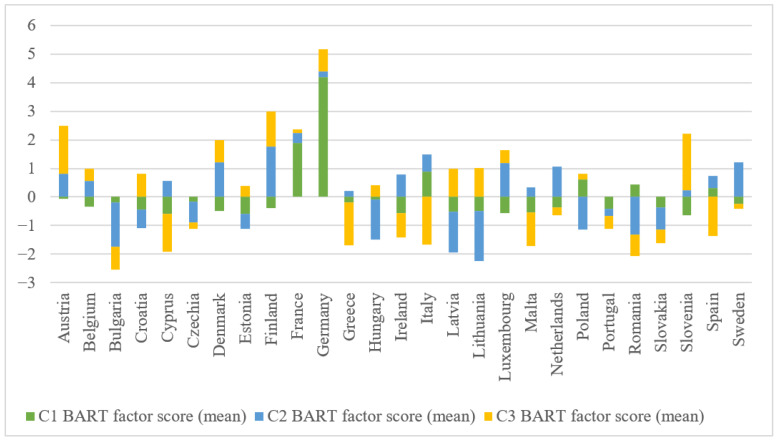
Average Bartlett scores for the three main components.

The results of the PCA factor analysis, summarized in the Bartlett mean scores for the three main components, allow for a differentiated interpretation of the psychosocial profile of each EU Member State. The results also highlight both the epidemiological characteristics of the mental burden and the level of functionality of the mental health system (Figure 3).

Component 1, institutionalized psychiatric burden, reflects the intensity of services used to treat mental disorders, particularly hospitalizations and specialized human resources. In this regard, according to the data represented graphically in Figure 3, Germany (score = 4.18) and France (1.88) stand out clearly from the other countries, indicating a high level of institutionalized diagnosis, robust hospital infrastructure, and a medical culture favourable to early intervention and formal psychiatric treatment.

These results are consistent with those of recent European comparative studies, which highlight the high level of hospital service utilization in Germany and France, associated with a predominantly biomedical mental health model, but also with a robust infrastructure of specialized human resources [42,57,115,116]. These countries are ranked first and second in Europe in terms of the number of patients discharged from hospital with a psychiatric diagnosis, with Germany exceeding the European average by a factor of ten and France by a factor of four.

In the case of Germany, this pattern reflects long-standing features of its mental health policy: a hospital-centred care model, comparatively slow progress in deinstitutionalisation, and sustained investment in psychiatric bed capacity rather than community-based services, all of which structurally reinforce the high institutionalized psychiatric burden captured by Component 1. Other countries with relevant positive scores are Italy (0.88), Poland (0.60), and Romania (0.42), which suggests a significant institutional burden on these systems due to the high prevalence of severe disorders.

The percentage of European averages exceeded for psychiatric patients discharged between 2014 and 2023 is as follows: Italy (130%), Poland (203%), and Romania (182%). At the opposite end of the spectrum, countries such as Slovenia (−0.64), Ireland (−0.55), Luxembourg (−0.57), and Estonia (−0.59) have negative scores, suggesting a less institutionalized approach or systemic underdiagnosis of mental disorders. In these countries, the national average ratio of discharged psychiatric patients to the European average is below 10%: Slovenia (9%), Ireland (5%), Luxembourg (5%), and Estonia (8%).

Component 2 (health system functionality and health perception) captures aspects related to the availability of general services, the perceived capacity of the health system, and the level of mental health education. Finland (1.77), Sweden (1.22), Denmark (1.20), Luxembourg (1.18), and the Netherlands (1.06) are significantly above average, reflecting well-performing, well-funded, and accessible health systems, as well as an advanced mental health culture. These positive scores can be linked to high levels of health literacy, strong prevention policies, and a digital infrastructure capable of supporting early interventions. In contrast, countries with significant negative scores, such as Lithuania (−1.76), Latvia (−1.41), Hungary (−1.39), Romania (−1.30), and Poland (−1.13), highlight persistent structural deficiencies, possible regional imbalances in access to treatment, and low levels of funding for mental health services.

Component 3 (suicidal vulnerability associated with addictive behaviours) integrates critical factors related to mortality associated with severe mental illness and the prevalence of problematic substance use (alcohol, drugs). Slovenia (1.98), Lithuania (1.02), Latvia (0.97), and Finland (1.23) have high scores, indicating acute psychosocial pressure and a worrying presence of suicide risks or addictive behaviours. These data are consistent with European statistics [116,117], showing that Baltic and Nordic countries have some of the highest suicide rates in the region, despite the quality of health services. On the other hand, Italy (−1.67), Greece (−1.51), and Spain (−1.37) stand out with negative scores, which may reflect protective cultural factors, stronger social networks, and a lower prevalence of self-harming behaviours.

This three-dimensional classification outlines a composite profile of mental health systems in the European Union, where not only the incidence of disorders but also the way in which they are organized, intervened, and prevented plays a crucial role in shaping national vulnerabilities.

There is also a partial decoupling between the degree of institutionalization (C1) and positive perceptions of the system (C2), as well as a divergence between systemic performance and suicide risk control (C3), suggesting the multidimensional complexity of mental health in the European context.

The crisis-comparative evidence presented by authors [116,117] provides an additional conceptual layer for interpreting our findings. Their analysis shows that psychiatric admissions undergo non-linear, crisis-driven reconfigurations when health systems are exposed to acute shocks such as natural disasters or pandemic lockdowns. These shifts do not merely reflect variations in clinical need but are the outcome of complex interactions between epidemiological pressure, institutional resilience, and the capacity of mental-health infrastructures to absorb sudden surges or disruptions in demand.

Seen through this lens, the latent dimensions identified in our PCA—particularly the institutionalized psychiatric burden and the suicidal vulnerability/addictive mortality axis—capture not only long-term structural differences between Member States but also the potential reactivity of these systems when confronted with large-scale emergencies. Integrating this crisis-sensitive perspective suggests that countries structurally positioned on the extremes of Components 1 or 3 may also be those whose institutional configurations are most susceptible to destabilization or adaptive strain under acute environmental or epidemiological stress.

Beyond the structural gaps identified in institutional and community-based care, the findings highlight the need for a coordinated European framework for monitoring community mental health services. As current Eurostat datasets do not systematically capture outpatient, mobile, crisis, or psychosocial support services, a structured observatory would require the development of harmonized definitions, minimum data standards, and multi-source reporting mechanisms. These considerations emerge directly from the factorial patterns and point to a clear methodological direction for future research.

### 4.2. Contextualizing Psychosocial Risk Patterns and Strategic Directions in European Mental Health Policies

The results obtained using principal component analysis reveal not only significant variations between EU Member States in terms of the prevalence, institutionalization, and treatment of mental disorders but also major discrepancies in the functional structure of health systems and their capacity to mitigate suicide risks and addictive consumption. These patterns cannot be analyzed in isolation; they must be integrated into a broader reading of the mental health strategies promoted at the European level and relevant transnational initiatives.

Over the last decade, the European Union has seen growing concern about mental health, with the recognition that mental illness is one of the leading causes of global disease burden [104,117,118,119,120,121], with a direct impact on productivity, social inclusion, and the sustainability of health systems. Programmes such as the EU Compass for Action on Mental Health and Well-being [31], Joint Action on Mental Health and Well-being [30], or initiatives related to EU4Health [32], and Mental Health in All Policies [122] have promoted systemic approaches based on prevention, intersectoral integration, and digitization of services. However, our analysis highlights that the implementation of these policies is uneven and that convergence among Member States in the field of mental health remains limited.

The recent literature [123,124,125,126] emphasizes that a positive perception of mental health does not necessarily guarantee effective control of suicide risks and that the perceived success of systems may mask gaps in community intervention and prevention of self-harming behaviours. Thus, while countries such as Finland, Sweden, and Luxembourg score high on Component 2, reflecting effective system functioning and a positive perception of mental health, this advantage does not automatically translate into effective control of suicidal vulnerability (Component 3). This aspect suggests the need for a balance between allocated resources and the effectiveness of interventions. Similarly, Germany and France, characterized by strong institutionalization (C1), fail to achieve equivalent performance in terms of prevention or systemic perception (C2). This may indicate a predominantly curative orientation to the detriment of community-based and multidisciplinary approaches.

On the other hand, Eastern European countries (Romania, Bulgaria, Latvia, Lithuania) continue to record negative values on all three components, outlining a systemic risk profile that involves both underfunding and underdiagnosis, persistent stigma, and the absence of integrated services. In these contexts, it is essential to strengthen institutional capacities, stimulate professional training in mental health, and expand community-based interventions.

The empirical findings generate a robust basis for the development of concrete policy recommendations that reflect and operationalise the latent structures revealed by the analysis. By integrating epidemiological, institutional, and behavioural indicators into a coherent framework, the analysis reveals systemic vulnerabilities that can be directly translated into differentiated policy actions. To ensure consistency and applicability, these recommendations are anchored in ongoing European strategies such as the EU4Health Programme, the European Mental Health Strategy (2023), and the WHO European Mental Health Framework for Action 2021–2025.

A first implication concerns the need to strengthen European cooperation in the monitoring of psychosocial risks. The heterogeneity observed in the institutional burden of psychiatric disorders demonstrates the lack of standardized indicators capable of capturing both prevalence and systemic response. Establishing harmonized monitoring tools and creating a permanent European observatory on psychosocial risks would enable more effective cross-country comparability, while also offering an evidence-based platform for coordinated action. This step is consistent with the EU4Health commitment to improving health data infrastructure and would substantially enhance the responsiveness of mental health governance at both national and EU levels.

A second recommendation emerges from the structural deficits in system functionality identified in several Eastern and Southern European countries. These results highlight the necessity of stimulating investment in prevention, digital health, and telepsychiatry, particularly in disadvantaged or rural regions where access to care remains severely constrained. Targeted funding for remote psychiatric services and digital platforms would not only reduce geographic inequalities but also align mental health care provision with the broader EU agenda for health system digitalization.

The European Mental Health Strategy 2023 explicitly calls for such transformations, emphasizing the importance of early intervention and the reduction in barriers to care through innovative service delivery models. Importantly, the high institutional burden observed in countries such as Germany and France suggest a potential over-reliance on hospital-based models of care, which may indirectly point to limited community coverage. While direct comparative data on community-based systems are unavailable in the Eurostat framework, this inference underscores the importance of strengthening outpatient and community-level alternatives, in line with existing WHO recommendations for scaling up primary mental health care.

Also, the elevated scores for suicidal vulnerability associated with substance use in certain Member States underscore the urgency of developing coordinated European guidelines for the integrated management of suicide, addictions, and severe psychiatric disorders. Current national responses remain fragmented, resulting in persistently elevated mortality rates and structural inefficiencies. A common European framework of guidelines, informed by successful examples such as the Nordic suicide prevention programmes or the integrated care networks in the Netherlands, would provide the necessary consistency across Member States and support convergence towards evidence-based standards of intervention.

These results highlight the critical importance of advancing longitudinal research on the social determinants of mental health. The PCA structure shows that psychosocial vulnerability cannot be understood solely through clinical or institutional variables but is closely intertwined with broader dimensions of social cohesion and sustainable development. Integrating mental health into the European Pillar of Social Rights, cohesion policies, and even the objectives of the European Green Deal would ensure that mental well-being is addressed as both a determinant and an outcome of sustainable growth. Such an approach would help shift policy paradigms, moving from reactive treatment to proactive governance that recognizes mental health as a cornerstone of resilience and equity in European societies.

### 4.3. Using Key Components to Build a European Mental Health Taxonomy

The answer to the first research question (How can these components and scores be used to construct a European taxonomy of mental health that allows for monitoring national performance and informing differentiated and effective public policies?*)* involves using the latent dimensions extracted using PCA as structural benchmarks for comparative analysis of mental health status and institutional response capacity within the European Union.

The identified components (C1—institutionalized psychiatric burden, C2—system functionality and health perception, C3—suicidal vulnerability and substance use) provide a coherent framework for interpreting the diversity of national mental health profiles. The proposal for a European taxonomy based on empirically extracted latent dimensions is aligned with current research directions promoting structural and multidimensional approaches to mental health, rather than one-dimensional assessments focused on prevalence or expenditure [127,128].

The standardized factor scores allow not only the ranking of Member States according to their relative position on these latent axes but also the delineation of regional clusters or typologies of risk and performance. Thus, the analysis of average scores shows that countries such as Germany and France have an institutionalized profile with solid infrastructure but a significant burden of severe mental disorders, while countries such as Finland and Denmark have high functional performance in terms of positive health perceptions and budget allocations but remain vulnerable on the suicidal dimension.

**Figure 4:**
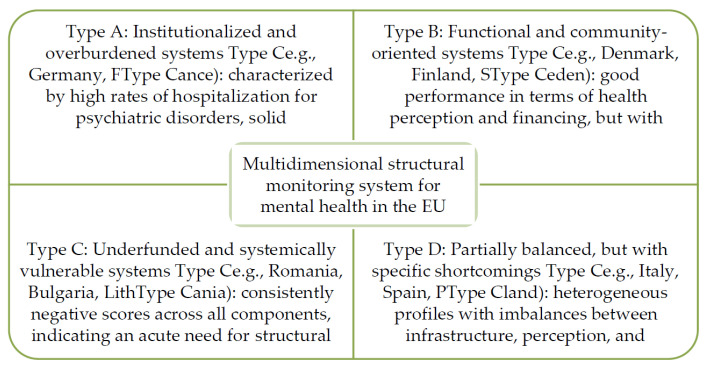
European taxonomy of mental health.

These components can form the basis of a multidimensional structural mental health monitoring system in the EU, replacing one-dimensional or strictly descriptive assessments with a synthetic, comparable, and intervention-oriented approach. Specifically, a European taxonomy framework can be developed that includes four categories of systems (Figure 4).

This taxonomy can be used as a diagnostic and benchmarking tool, both for prioritizing investments in infrastructure and personnel and for developing differentiated prevention and intervention policies, depending on the structural specificities of each country. Furthermore, the regular integration of new Eurostat data into this factorial architecture would allow for longitudinal monitoring, reflecting the effectiveness of interventions, risk trajectories, and the progress or regression of national mental health policies.

### 4.4. Implications for Institutional Reform and Mental Health Strategies in Europe

The answer to the second research question—What are the implications of these results regarding the need for institutional reform and future strategies for prevention, early intervention, and destigmatization in the field of mental health?—calls for a translational interpretation of the conclusions of the factor analysis towards reforming current mental health governance paradigms. The findings highlight profound structural asymmetries between EU Member States. These results suggest a critical reassessment of existing strategies and a contextual adaptation of intervention measures to the psycho-epidemiological specificities of each national system.

High scores on Component 1 (institutionalized psychiatric burden in countries such as Germany, France, and Italy) suggest intensive use of hospital resources for the management of severe mental disorders, indicating over-medicalization of the phenomenon to the detriment of early community interventions and effective outpatient services. Recent reforms recommend rebalancing mental health systems by reducing dependence on hospital treatment and strengthening community, digital, and preventive services [43,117,129,130,131,132]. In this context, a recalibration of the service delivery model is needed, through a gradual shift towards patient-centred approaches, in which prevention and early intervention (including in schools and workplaces) are central pillars of intervention. Models validated in Nordic countries (e.g., Finland, Sweden), which combine adequate funding with integrated mental health networks, can be adapted and replicated in other national contexts.

At the same time, Component 2, health system functionality and health perception, reflects a significant association between system resources (expenditure, infrastructure, density of specialized staff) and subjective health status. The disparities reported between countries with robust infrastructure and those with chronic deficits, such as Romania, Bulgaria, and Lithuania, call for institutional strengthening interventions. Such interventions should focus on increasing early diagnosis capacity, reducing waiting times, and continuing training for mental health personnel. At the same time, legislative reform is needed to ensure equitable access to services by extending insurance coverage and reducing financial and geographical barriers.

Component 3, suicidal vulnerability associated with addictive behaviours, highlights the profound psychosocial dimension of mental health, which is linked not only to clinical factors but also to structural determinants, such as economic precariousness, social isolation, and a lack of support networks. The results obtained indicate the need for a multisectoral approach, integrating mental health interventions into social, educational, and employment policies, with a focus on preventing suicidal behaviour and problematic alcohol and drug use. Stigma reduction campaigns need to be strengthened and anchored in communities, involving the media, local leaders, patient associations, and educators in promoting a culture of mental health as an essential dimension of individual and collective well-being.

This research stands out for its innovative approach to mental health analysis at the European level, using principal component analysis (PCA) to identify latent dimensions of psychosocial vulnerability and the functionality of mental health systems. This multidimensional approach, empirically grounded in nationally relevant and European-harmonized indicators, offers a rigorous alternative to traditional one-dimensional perspectives, which fail to capture the real complexity of contemporary psychiatric phenomena. The relevance of this research is supported by the convergence of two major trends in the European public sphere: on the one hand, the growing recognition of the global burden of mental disorders, accentuated by recent crises (the COVID-19 pandemic, socioeconomic instability, migration, and conflict), and, on the other hand, the emergence of strategic European initiatives aimed at integrating mental health into cross-cutting public policies. In this context, the results obtained contribute to a nuanced understanding of the position of EU Member States in relation to three fundamental dimensions (psychiatric institutionalization, system perception, and suicidal/addictive vulnerability) facilitating the formulation of differentiated and contextualized interventions.

The novelty of the approach lies in the articulation of a European typology of mental health, based on standardized factor scores. This allows not only the ranking of national systems according to performance and risk but also the definition of operational paradigms for longitudinal monitoring and comparative evaluation.

To support the development of a future European observatory for community mental health services, several methodological priorities are outlined that can guide the next stages of research. First, a harmonized conceptual framework is required, defining core dimensions of community-based care (such as accessibility, continuity, deinstitutionalization intensity, integration with primary care, mobile crisis response, and rehabilitation). Second, standardized indicators must be developed to capture these dimensions, including service penetration rates, caseload structures, staffing profiles, crisis pathway activation, and the balance between inpatient and outpatient care. Third, data collection should move beyond national aggregates by incorporating regional disaggregation and multi-source architectures that combine administrative registers, health information systems, social care datasets, and population-based surveys. Fourth, pilot testing should be undertaken in selected Member States to validate indicator feasibility, comparability, and temporal stability before scaling to a pan-European platform.

Together, these methodological steps provide a realistic and actionable roadmap for building a coherent monitoring infrastructure capable of complementing and refining the structural patterns identified in this factorial analysis.

The findings of this study hold distinct relevance for both academic inquiry and policy development.

For researchers, the proposed model offers a standardized and replicable basis for multivariate analysis of mental health, providing an empirical framework that can be extended through longitudinal designs or validated in non-European contexts. By integrating epidemiological, institutional, and behavioural indicators into a coherent latent structure, the model facilitates cross-national comparisons while also enabling the exploration of systemic dynamics that remain obscured in unidimensional approaches.

For policymakers, the tripartite structure functions as a rapid diagnostic tool that differentiates between domains of institutional burden, system functionality, and acute psychosocial vulnerability. This differentiation has direct practical implications. It allows interventions to be tailored to specific risk profiles. For example, countries with high scores on the vulnerability dimension should prioritize suicide prevention and addiction programmes. Under-resourced systems would benefit from investments in infrastructure and human resources. In states that rely excessively on hospital-based psychiatric care, reforms should focus on advancing deinstitutionalization. In this sense, the taxonomy derived from the factor analysis not only enhances theoretical understanding but also translates into actionable guidance for the design of equitable and context-sensitive mental health strategies.

The taxonomy developed in this study can be operationalised in several concrete ways to support policymaking. First, cluster membership can guide the prioritization of targeted interventions: countries situated in the high-vulnerability cluster (characterized by elevated suicide, alcohol- and drug-related mortality) may prioritize suicide-prevention strategies, early intervention services, and integrated addiction-treatment programmes. Second, Member States positioned within clusters dominated by institutionalized psychiatric burden can use the taxonomy to justify reallocating resources toward community-based mental health services, crisis-response teams, and workforce expansion in outpatient psychiatry. Third, systems belonging to clusters with lower perceived health and reduced expenditure can leverage these insights to strengthen primary care integration, increase preventive investment and address broader social determinants of mental health. Through such differentiated approaches, the taxonomy provides a practical framework for aligning resource allocation and intervention design with the specific structural vulnerabilities identified across the EU.

Taken together, these crisis-sensitive insights further consolidate the contribution of the present study to European psychiatric epidemiology and public health governance. By demonstrating that the latent structures identified through PCA are consistent with crisis-driven fluctuations in psychiatric demand, the analysis highlights the need for governance models capable of integrating both long-term structural determinants and the acute reactivity of mental-health systems. This reinforces the value of a multilevel approach in which institutional resources, behavioural vulnerabilities, and systemic resilience are jointly assessed. In this sense, the empirical taxonomy proposed here provides a meaningful framework for European policy-makers seeking to design more adaptive, equitable, and evidence-based strategies for mental-health planning.

## 5. Conclusions

In this study, we propose a comprehensive and methodologically sound analysis of psychosocial profiles in the European Union, using an advanced multivariate approach, principal component analysis (PCA), applied to an extensive set of epidemiological, institutional, and behavioural indicators of mental health. By investigating 27 Member States over the 2014–2023 period, we succeed in capturing the complexity of the institutionalized determinants of mental health at the population level and propose a differentiated mapping of systemic risks and resources.

The three components extracted—(1) the intensity of the institutionalized psychiatric burden, (2) the perception and efficiency of the health care system, and (3) suicidal vulnerability associated with addictive behaviours—synthesize the latent structure of European mental health in a coherent and statistically valid manner.

Based on average factor scores, it was possible to empirically classify Member States according to their specific patterns of psychosocial risk and institutional response capacity, providing the basis for the construction of a European taxonomy of mental health. This typology, in relation to the first research question (Q1), validates the usefulness of PCA scores as a robust tool for comparative monitoring and strategic planning of interventions in the field of community psychiatry. Regarding the second research question (Q2), the results convincingly reveal the need for a profound and differentiated reform of mental health systems. Such reforms are especially needed in countries with high scores associated with excessive institutionalization, poor access to health services, and high rates of suicide and problematic alcohol or drug use.

In this context, it is imperative to reconfigure the intervention paradigm by focusing on prevention, early identification, expanding outpatient services, and continued digitization of psychiatric infrastructure. At the same time, combating stigma through educational interventions and promoting mental health in the community and in schools are priority areas for public policy in this field, with a transgenerational impact.

Although the results are statistically significant and relevant, several limitations must be acknowledged. Beyond the constraints already discussed, a further methodological caveat concerns the potential influence of population size bias on the country-specific patterns identified in the taxonomy.

As the analysis uses standardized absolute counts for several hospitalization indicators, countries with large populations (such as Germany or France) may appear more prominently within certain components. While the standardization applied prior to PCA mitigates scale-related discrepancies, it cannot completely remove residual population effects. Sensitivity checks based on per capita indicators were not possible for all variables due to incomplete harmonization of population-adjusted metrics in Eurostat. This limitation has been explicitly recognized, and the interpretation of country-level factor scores should therefore be made with caution, particularly for high-population Member States.

Future research will benefit from replicating the analysis using population-adjusted or age-standardized indicators once harmonized and complete rate-based datasets become available across all diagnostic categories and years. The database spans ten years but is aggregated at the national level, which prevents capturing regional heterogeneity or the specific situation of vulnerable groups (e.g., youth, the elderly, or migrants).

The analysis is based on a balanced panel of 270 country–year observations, yet the use of pooled PCA primarily captures a cross-sectional latent structure. As such, the method identifies correlations among indicators but cannot establish causality or reveal temporal dynamics. Moreover, the factorial solution depends on the sample and period studied, which limits its predictive value and calls for replication in other contexts. Additional caveats include the absence of an external dataset or confirmatory factor analysis, which underlines the exploratory character of this study, and the exclusive reliance on objective structural indicators, without incorporating subjective psychosocial dimensions, such as stigma, service satisfaction, or treatment effectiveness. In future research, the factorial structure identified through PCA could be formally tested using confirmatory factor analysis (CFA). Such an approach would allow the specification of a measurement model aligned with the three latent dimensions extracted here, providing model-fit indices and validating the coherence of the proposed taxonomy. Additionally, a structural equation modelling (SEM) framework could further examine directional pathways between latent components and systemic characteristics, offering a more comprehensive confirmatory structure beyond the exploratory scope of PCA. At the same time, because this study is based on national-level aggregates, there is an inherent risk of ecological fallacy: associations observed at the country level cannot be generalized to individuals or social groups.

A further limitation stems from the incomplete availability of harmonized indicators for community-based mental health services in Eurostat. The proposal outlined in this study for establishing a European observatory for community mental health services would therefore require a clearer methodological roadmap.

Future research should prioritize: (1) the development of standardized operational definitions for community mental health provision, (2) the construction of comparable indicators capturing service accessibility, intensity, continuity, and integration with primary care, (3) the collection of disaggregated regional data to overcome the limits of national-level aggregation, and (4) piloting a multi-source architecture combining administrative registers, health information systems, and population-based surveys.

Such a framework would support the long-term objective of creating a harmonized European monitoring platform capable of validating, complementing, and refining the indirect patterns identified through the present factorial analysis.

In practical terms, the three-component structure derived from our analysis provides a concise yet actionable framework for both research and policy. For scholars, it establishes a replicable basis for longitudinal and comparative studies of mental health systems. For policymakers, it offers a diagnostic tool that can guide differentiated strategies—whether by prioritizing suicide prevention where vulnerability is acute, investing in infrastructure where systemic deficits persist, or promoting deinstitutionalization where over-medicalization dominates.

The taxonomy developed translates complex statistical results into clear orientations for evidence-based and context-sensitive mental health policies. Future research will prioritize the development of harmonized indicators of community mental health services, in order to validate and refine the indirect patterns identified here.

Although the factorial structure extracted in this study is robust and stable across the ten-year period, an important methodological limitation concerns the absence of external validation using independent datasets such as the WHO or Global Burden of Disease indicators. These sources include measures of disability, prevalence, and years lived with disability that could complement the Eurostat-based structural indicators and enable a cross-system comparison of latent dimensions. However, the heterogeneous temporal coverage, differences in diagnostic framings, and incomplete alignment with Eurostat categories limit the feasibility of integrating such datasets into the present model without compromising the comparability and balance of the panel.

Similarly, while confirmatory factor analysis or structural equation modelling would offer a more formal assessment of construct validity, these techniques require strong a priori assumptions regarding the covariance structure, as well as a substantially larger number of observations per latent factor than the one afforded by the EU-27 panel. Incorporating a full CFA/SEM framework would considerably expand the scope of the manuscript and shift its focus toward model-driven hypothesis testing rather than exploratory structural identification. Accordingly, the analysis remains exploratory, while acknowledging that the validation of the taxonomy should be pursued in future research through externally sourced indicators and confirmatory modelling techniques, contingent on the availability of harmonized multi-source datasets.

A further structural limitation arises from the absence of standardized indicators on community-based mental health services within Eurostat, which restricts the capacity of this analysis to capture dimensions such as outpatient coverage, accessibility, continuity of care, or service responsiveness. The dataset provides a detailed view of hospital-based activity and mortality patterns, yet it remains largely silent on the performance of ambulatory psychiatric services, early intervention teams, mobile crisis units, or rehabilitation programmes, elements that are increasingly recognized by the WHO and OECD as central to modern mental health systems. This lack of community-level metrics prevents a balanced assessment of the institutional–community continuum and may obscure heterogeneity in pathways of care across Member States.

Future research would benefit from the development of a harmonized European observatory capable of integrating indicators such as outpatient care coverage rates, waiting times for psychiatric consultations, frequency of follow-up contacts after hospital discharge, and indicators of continuity and coordination of care. Incorporating such measures would enhance the granularity and policy relevance of comparative analyses and allow the identification of systemic gaps that remain invisible within hospital-centred datasets.

Despite these limitations, this research offers an original, current, and relevant contribution to the specialized literature and to decision-makers involved in mental health governance. By establishing a tripartite factorial structure and using it to construct a differentiated European taxonomy, the work expands the research horizon toward a more coherent framework for monitoring, intervention planning, and strategic resource allocation. In a European context marked by overlapping crises (health, economic, and demographic), mental health is becoming a synthetic indicator of social cohesion and the sustainability of public health systems. From this perspective, the future extension of the analysis to longitudinal dimensions, the integration of individual data, and mixed quantitative–qualitative exploration emerges as essential directions for refining strategies and strengthening psychosocial resilience at the European level.

## Figures and Tables

**Table 2:** KMO and Bartlett’s test.

Kaiser–Meyer–Olkin Measure of Sampling Adequacy		0.747
Bartlett’s Test of Sphericity	Approx. Chi-Square	9259.266
	df	153
	Sig.	0.000

**Table 3:** Communalities (extraction method: principal component analysis).

Variable	Initial	Extraction	Variable	Initial	Extraction	Variable	Initial	Extraction
DT	1.000	0.888	DSUICID	1.000	0.716	EXTPMENTAL	1.000	0.963
LEX	1.000	0.707	DALC	1.000	0.594	EXTPDEMENT	1.000	0.595
PGPH	1.000	0.607	DSNERV	1.000	0.449	EXTPALC	1.000	0.914
CHE	1.000	0.762	TSPEC	1.000	0.872	EXTPDRUG	1.000	0.829
HBTI	1.000	0.699	PSIHSPEC	1.000	0.937	EXTPSCHIZ	1.000	0.859
DDRUG	1.000	0.685	EXTPT	1.000	0.982	EXTPAFECT	1.000	0.935

**Table 4:** Total variance explained (extraction method: principal component analysis).

Component	Initial Eigenvalues			Extraction Sums of Squared Loadings			Rotation Sums of Squared Loadings		
	Total	% of Variance	Cumulative %	Total	% of Variance	Cumulative %	Total	% of Variance	Cumulative %
1	8.910	49.501	49.501	8.910	49.501	49.501	8.855	49.196	49.196
2	3.113	17.294	66.794	3.113	17.294	66.794	3.089	17.159	66.355
3	1.971	10.949	77.744	1.971	10.949	77.744	2.050	11.389	77.744
4	0.887	4.925	82.669	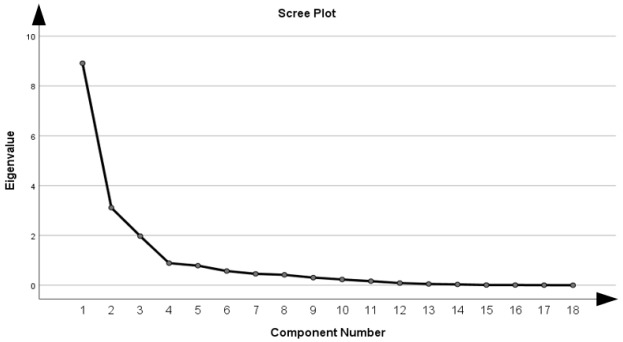					
5	0.784	4.356	87.024						
6	0.570	3.165	90.190						
7	0.457	2.540	92.730						
8	0.418	2.323	95.053						
9	0.306	1.698	96.751						
10	0.231	1.284	98.035						
11	0.162	0.900	98.935						
12	0.087	0.484	99.420						
13	0.051	0.282	99.702						
14	0.032	0.179	99.881						
15	0.009	0.052	99.932						
16	0.008	0.045	99.977						
17	0.004	0.022	99.999						
18	0.000	0.001	100.000						

**Table 5:** Rotated component matrix (extraction method: principal component analysis).

Variable	C1—Institutionalized Psychiatric Burden	C2—Health System Functionality and Health Perception	C3—Suicidal Vulnerability Associated with Addictive Behaviours
EXTPT	0.991	0.011	−0.015
EXTPMENTAL	0.974	0.004	0.123
EXTPAFECT	0.961	0.021	0.107
PSIHSPEC	0.956	0.147	−0.041
EXTPALC	0.937	0.004	0.188
DT	0.929	0.027	−0.155
EXTPSCHIZ	0.925	−0.058	0.013
TSPEC	0.913	0.079	−0.177
EXTPDRUG	0.896	0.070	0.143
EXTPDEMENT	0.766	−0.031	0.084
CHE	0.185	0.833	0.182
LEX	0.124	0.824	−0.114
HBTI	0.384	−0.687	0.280
PGPH	0.008	0.647	−0.433
DSNERV	−0.046	0.634	0.213
DALC	0.029	0.089	0.765
DSUICID	−0.090	−0.373	0.754
DDRUG	0.240	0.487	0.625

**Table 6:** Initial Cluster Centres.

Variable	Cluster Centre 1	Cluster Centre 2	Cluster Centre 3
REGR factor score 1 for analysis 1	4.35172	−0.64020	0.91305
REGR factor score 2 for analysis 1	0.07757	1.00930	0.65024
REGR factor score 3 for analysis 1	1.15383	2.92396	−1.83183

**Table 7:** Iteration History.

Iteration ^a^	Change in Cluster Centres		
	1	2	3
1	0.584	2.035	1.738
2	0.487	0.121	0.114
3	0.000	0.083	0.068
4	0.000	0.048	0.040
5	0.000	0.021	0.017
6	0.000	0.044	0.036
7	0.000	0.023	0.019
8	0.000	0.031	0.026
9	0.000	0.000	0.000

^a^ Convergence achieved due to no or small change in cluster centres. The maximum absolute coordinate change for any centre is 000. The current iteration is 9. The minimum distance between initial centres is 4.590.

**Table 8:** Final Cluster Centres.

Variable	Cluster Centre 1	Cluster Centre 2	Cluster Centre 3
REGR factor score 1 for analysis 1	3.49545	−0.35191	−0.11026
REGR factor score 2 for analysis 1	0.22935	−0.11828	0.07337
REGR factor score 3 for analysis 1	0.58482	0.87855	−0.81218

## Data Availability

The data presented in this study are available on request from the corresponding author due to the fact that the original Eurostat datasets were pre-processed, aggregated, and reformatted for the purposes of the analysis. The processed datasets used in this article can be provided upon reasonable request.

## Appendix A

**Table A1:** Yearly KMO and Bartlett’s Test.

Test/Year		2014	2015	2016	2017	2018	2019	2020	2021	2022	2023
Kaiser-Meyer-Olkin Measure of Sampling Adequacy.		0.723	0.717	0.722	0.704	0.715	0.698	0.680	0.663	0.596	0.553
Bartlett’s Test of Sphericity	Approx. Chi-Square	779.385	809.399	797.592	790.304	805.359	802.723	783.901	780.962	812.230	818.990
	df	153.000	153.000	153.000	153.000	153.000	153.000	153.000	153.000	153.000	153.000
	Sig.	0.000	0.000	0.000	0.000	0.000	0.000	0.000	0.000	0.000	0.000

**Table A2:** Annual stability analysis of communalities (2014–2023).

Variables	Initial	Extraction									
		2014	2015	2016	2017	2018	2019	2020	2021	2022	2023
DT	1.000	0.899	0.898	0.895	0.888	0.907	0.911	0.874	0.888	0.884	0.883
LEX	1.000	0.705	0.730	0.712	0.721	0.712	0.741	0.770	0.768	0.760	0.748
PGPH	1.000	0.662	0.653	0.656	0.684	0.673	0.663	0.613	0.501	0.480	0.483
CHE	1.000	0.866	0.871	0.852	0.843	0.810	0.770	0.738	0.703	0.709	0.691
HBTI	1.000	0.750	0.725	0.702	0.693	0.699	0.709	0.684	0.695	0.675	0.680
DDRUG	1.000	0.604	0.698	0.745	0.748	0.710	0.677	0.626	0.681	0.691	0.731
DSUICID	1.000	0.685	0.755	0.731	0.735	0.786	0.737	0.761	0.698	0.758	0.794
DALC	1.000	0.590	0.614	0.639	0.524	0.538	0.534	0.557	0.646	0.690	0.682
DSNERV	1.000	0.428	0.382	0.384	0.454	0.467	0.465	0.486	0.483	0.502	0.482
TSPEC	1.000	0.899	0.898	0.894	0.905	0.856	0.868	0.857	0.862	0.861	0.865
PSIHSPEC	1.000	0.907	0.901	0.897	0.900	0.958	0.961	0.962	0.961	0.963	0.962
EXTPT	1.000	0.979	0.979	0.978	0.979	0.990	0.990	0.989	0.989	0.984	0.982
EXTPMENTAL	1.000	0.946	0.948	0.951	0.952	0.976	0.976	0.976	0.975	0.975	0.973
EXTPDEMENT	1.000	0.596	0.558	0.558	0.539	0.614	0.612	0.613	0.606	0.660	0.684
EXTPALC	1.000	0.916	0.916	0.922	0.925	0.920	0.919	0.919	0.918	0.930	0.925
EXTPDRUG	1.000	0.835	0.847	0.855	0.855	0.814	0.819	0.823	0.828	0.815	0.823
EXTPSCHIZ	1.000	0.873	0.876	0.879	0.881	0.874	0.867	0.859	0.861	0.891	0.885
EXTPAFECT	1.000	0.902	0.903	0.908	0.906	0.958	0.957	0.957	0.954	0.960	0.953

Extraction Method: Principal Component Analysis.

**Table A3:** Annual Variance Explained (2014–2023).

Component/Year	Extraction Sums of Squared Loadings (% of Variance)									
	2014	2015	2016	2017	2018	2019	2020	2021	2022	2023
1	49.09	49.32	49.73	49.36	50.72	50.05	49.59	49.67	50.28	50.3
2	18.56	18.38	18.21	18.48	17.48	17.55	17.55	16.44	15.52	15.32
3	10.36	10.91	10.72	10.69	11.02	11.16	10.98	11.77	13.02	13.4
Total	78.01	78.61	78.66	78.53	79.22	78.76	78.12	77.88	78.82	79.02
**Component/Year**	**Rotation Sums of Squared Loadings (% of Variance)**									
	**2014**	**2015**	**2016**	**2017**	**2018**	**2019**	**2020**	**2021**	**2022**	**2023**
1	48.36	48.65	49.1	48.87	50.23	49.65	49.45	49.52	50.16	50.05
2	17.97	18.39	18.25	17.85	17.17	16.74	17.37	16.32	15.16	15.39
3	11.69	11.58	11.32	11.8	11.83	12.36	11.32	12.04	13.5	13.58
Total	78.02	78.62	78.67	78.52	79.23	78.75	78.14	77.88	78.82	79.02

**Table A4:** Rotated Component Matrix (2014–2023).

***Variable***		***All Years***	**2014**	**2015**	**2016**	**2017**	**2018**	**2019**	**2020**	**2021**	**2022**	**2023**
EXTPT	*Component 1*	*0.991*	0.988	0.989	0.989	0.989	0.995	0.995	0.994	0.994	0.991	0.991
EXTPMENTAL		*0.974*	0.959	0.959	0.960	0.965	0.981	0.980	0.982	0.981	0.983	0.980
EXTPAFECT		*0.961*	0.939	0.938	0.940	0.943	0.973	0.971	0.973	0.972	0.976	0.972
PSIHSPEC		*0.956*	0.937	0.935	0.933	0.930	0.965	0.966	0.970	0.970	0.972	0.973
EXTPALC		*0.937*	0.931	0.930	0.932	0.939	0.942	0.940	0.943	0.941	0.954	0.946
DT		*0.929*	0.937	0.935	0.933	0.924	0.940	0.940	0.918	0.932	0.931	0.931
EXTPSCHIZ		*0.925*	0.925	0.927	0.929	0.932	0.934	0.931	0.926	0.927	0.943	0.940
TSPEC		*0.913*	0.930	0.929	0.926	0.925	0.903	0.905	0.900	0.913	0.912	0.916
EXTPDRUG		*0.896*	0.895	0.899	0.900	0.908	0.888	0.891	0.896	0.897	0.896	0.898
EXTPDEMENT		*0.766*	0.741	0.728	0.743	0.728	0.781	0.775	0.776	0.771	0.807	0.820
CHE		*0.185*	0.145	0.161	0.166	0.170	0.195	0.185	0.196	0.189	0.225	0.209
LEX		*0.124*	0.144	0.112	0.120	0.102	0.132	0.132	0.104	0.131	0.143	0.141
HBTI		*0.384*	0.357	0.370	0.386	0.407	0.408	0.399	0.384	0.375	0.367	0.358
PGPH		*0.008*	0.009	0.011	0.036	0.036	0.026	−0.001	−0.035	−0.028	−0.022	0.018
DSNERV		*−0.046*	−0.046	−0.040	−0.043	−0.065	−0.039	−0.055	−0.049	−0.055	−0.051	−0.039
DALC		*0.029*	0.030	0.016	0.001	0.064	0.037	0.037	0.011	0.019	−0.004	0.048
DSUICID		*−0.090*	−0.122	−0.165	−0.153	−0.100	−0.121	−0.075	−0.078	−0.063	−0.024	−0.003
DDRUG		*0.240*	0.217	0.320	0.378	0.309	0.337	0.205	0.224	0.163	0.141	0.134
EXTPT	*Component 2*	*0.011*	0.028	0.016	0.014	0.032	0.018	0.016	−0.001	−0.002	0.006	0.015
EXTPMENTAL		*0.004*	−0.079	−0.064	−0.050	0.022	0.028	0.058	0.021	0.010	0.030	0.009
EXTPAFECT		*0.021*	−0.062	−0.050	−0.036	0.029	0.045	0.074	0.039	0.026	0.046	0.029
PSIHSPEC		*0.147*	0.157	0.161	0.165	0.170	0.157	0.145	0.138	0.136	0.113	0.120
EXTPALC		*0.004*	−0.082	−0.053	−0.037	0.055	0.021	0.069	0.021	0.006	0.026	−0.019
DT		*0.027*	0.101	0.080	0.070	0.025	0.027	−0.003	−0.008	−0.033	−0.019	0.036
EXTPSCHIZ		*−0.058*	−0.122	−0.129	−0.130	−0.113	−0.020	−0.014	−0.039	−0.040	−0.021	−0.025
TSPEC		*0.079*	0.148	0.123	0.115	0.063	0.078	0.043	0.044	0.064	0.024	0.055
EXTPDRUG		*0.070*	−0.003	0.005	0.015	0.090	0.083	0.120	0.087	0.079	0.065	0.034
EXTPDEMENT		*−0.031*	−0.015	0.014	0.013	0.047	−0.010	−0.020	−0.074	−0.098	−0.021	−0.029
CHE		*0.833*	0.679	0.757	0.788	0.894	0.863	0.858	0.832	0.813	0.810	0.794
LEX		*0.824*	0.789	0.838	0.833	0.799	0.813	0.785	0.858	0.862	0.821	0.841
HBTI		*−0.687*	−0.776	−0.737	−0.716	−0.587	−0.648	−0.581	−0.681	−0.706	−0.694	−0.724
PGPH		*0.647*	0.813	0.799	0.773	0.620	0.644	0.539	0.644	0.558	0.354	0.505
DSNERV		*0.634*	0.497	0.540	0.604	0.668	0.663	0.669	0.660	0.655	0.672	0.619
DALC		*0.089*	−0.107	−0.050	−0.036	0.200	0.118	0.258	0.065	0.028	0.070	0.037
DSUICID		*−0.373*	−0.726	−0.652	−0.586	−0.276	−0.300	−0.135	−0.206	−0.235	−0.087	−0.132
DDRUG		*0.487*	0.106	0.245	0.287	0.651	0.514	0.670	0.547	0.445	0.554	0.479
EXTPT	*Component 3*	*−0.015*	0.036	0.022	0.023	−0.027	−0.014	−0.027	−0.028	−0.016	−0.037	−0.021
EXTPMENTAL		*0.123*	0.140	0.155	0.162	0.141	0.116	0.111	0.109	0.111	0.089	0.111
EXTPAFECT		*0.107*	0.133	0.145	0.152	0.123	0.099	0.091	0.093	0.088	0.071	0.092
PSIHSPEC		*−0.041*	0.065	0.024	0.009	−0.073	−0.041	−0.082	−0.048	−0.039	−0.070	−0.042
EXTPALC		*0.188*	0.207	0.219	0.225	0.202	0.180	0.173	0.172	0.182	0.140	0.173
DT		*−0.155*	−0.105	−0.136	−0.139	−0.186	−0.149	−0.166	−0.176	−0.133	−0.132	−0.121
EXTPSCHIZ		*0.013*	−0.042	−0.017	−0.012	0.016	0.015	0.027	0.027	0.021	0.040	0.038
TSPEC		*−0.177*	−0.112	−0.144	−0.150	−0.213	−0.185	−0.216	−0.211	−0.154	−0.169	−0.153
EXTPDRUG		*0.143*	0.188	0.197	0.210	0.154	0.134	0.104	0.113	0.129	0.089	0.120
EXTPDEMENT		*0.084*	0.216	0.166	0.078	0.083	0.068	0.104	0.068	0.042	0.095	0.105
CHE		*0.182*	0.620	0.521	0.451	0.125	0.166	−0.012	0.084	0.084	−0.046	0.130
LEX		*−0.114*	0.247	0.119	0.062	−0.269	−0.183	−0.326	−0.151	−0.091	−0.257	−0.146
HBTI		*0.280*	0.140	0.214	0.201	0.428	0.336	0.461	0.270	0.235	0.243	0.164
PGPH		*−0.433*	0.022	−0.122	−0.239	−0.546	−0.507	−0.610	−0.444	−0.435	−0.595	−0.477
DSNERV		*0.213*	0.423	0.298	0.131	0.063	0.163	0.121	0.219	0.226	0.217	0.312
DALC		*0.765*	0.760	0.782	0.798	0.693	0.723	0.683	0.744	0.803	0.828	0.823
DSUICID		*0.754*	0.379	0.550	0.604	0.806	0.825	0.845	0.844	0.799	0.866	0.881
DDRUG		*0.625*	0.739	0.732	0.721	0.479	0.576	0.431	0.526	0.676	0.603	0.696

Extraction Method: Principal Component Analysis. Rotation Method: Varimax with Kaiser Normalization. Rotation converged in 5 iterations. This table is a histogram representation designed to illustrate the temporal variations in Pearson correlation coefficients. The use of color serves as a visual encoding of the magnitude and direction of these coefficients, thereby facilitating an immediate and intuitive interpretation of the data.

**Table A5:** Cluster typology of EU Member States based on latent mental health system dimensions.

Cluster	Member States	Characteristic Profile	Avg. Distance
**Cluster 1**	Germany	High institutionalization combined with strong specialist capacity; distinct outlier with highly concentrated inpatient care	0.875
**Cluster 2**	Austria, Belgium, Croatia, Denmark, Estonia, Finland, France, Hungary, Latvia, Lithuania, Luxembourg, Poland, Slovenia	Mixed systems with moderate-to-high institutional reliance and robust health system capacity; heterogeneous resource distribution	1.148 (general mean)
**Cluster 3**	Bulgaria, Cyprus, Czechia, Greece, Ireland, Italy, Malta, Netherlands, Portugal, Romania, Slovakia, Spain, Sweden	Lower reliance on institutional care and higher exposure to psychosocial mortality risks; relatively diversified structural patterns	~1.03 (approx.)
